# Supplementary material for: Replacing conventional battery electrolyte additives with dioxolone derivatives for high-energy-density lithium-ion batteries
Source: Nat Commun. 2021 Feb 5;12:838. doi: 10.1038/s41467-021-21106-6 (PMC7864909; doi:10.1038/s41467-021-21106-6)
Supplement: Supplementary file 1 — Supplementary Information [file 41467_2021_21106_MOESM1_ESM.pdf]

Supplementary Information

Replacing conventional battery electrolyte additives with dioxolone derivatives for high-energy-density lithium-ion batteries

Sewon Park[1], Seo Yeong Jeong[2], Tae Kyung Lee[1,3], Min Woo Park[1], Hyeong Yong Lim[1], Jaekyung Sung[1], Jaephil Cho[1], Sang Kyu Kwak[1,*], Sung You Hong[2,*] and Nam-Soon Choi[1,*]

[1]School of Energy and Chemical Engineering, Ulsan National Institute of Science and Technology (UNIST), 50 UNIST-gil, Ulsan 44919, Republic of Korea

[2]Department of Chemistry, Ulsan National Institute of Science and Technology (UNIST), 50 UNIST-gil, Ulsan 44919, Republic of Korea

[3]Photovoltaics Research Department, Korea Institute of Energy Research (KIER), 152 Gajeong-ro, Yuseong-gu, Daejeon 34129, Republic of Korea

***Corresponding authors**

e-mail: skkwak@unist.ac.kr; syhong@unist.ac.kr; nschoi@unist.ac.kr

Supplementary methods

Optimization of reaction conditions

To establish the optimal reaction conditions, we investigated the various reaction parameters for the preparation of DMVC-OCF_3 and DMVC-OTMS (see also Supplementary Table 1).

DMVC-OCF_3: Under standard conditions, DMVC-OCF_3 was isolated in 45% yield (entry A1). No product formation was observed in the absence of $TMSCF_3$ or AgOTf (entries A2-4). The use of CsF instead of KF was less effective (entry A5). Reduced temperature and decreased reaction time yielded the product in lower yields (entries A6, A7).

DMVC-OTMS: The standard conditions provided DMVC-OTMS in 87% isolated yield (entry B1). In the absence of TMSCl or imidazole, no product formation was observed (entries B2, B3). TMSBr was not as efficient as TMSCl (entry B4). TEA as a base instead of imidazole gave a lower isolated yield (entry B5). The reaction yield was not significantly different at 50 °C (entry B6). A reduction in the reaction time (6 h) led to diminished yield (79%) (entry B7).

[**Condition A**]: To a mixture of AgOTf (1.03 g, 4.0 mmol, 2.0 equiv), Selectfluor (1.06 g, 3.0 mmol, 1.5 equiv), KF (350 mg, 6.0 mmol, 3.0 equiv), and DMVC-OH (260 mg, 2 mmol, 1.0 equiv) in ethyl acetate (10 mL) were added 2-fluoropyridine (388 mg, 4.0 mmol, 2.0 equiv) and $TMSCF_3$ (569 mg, 4.0 mmol, 2.0 equiv) under an argon atmosphere. Stirring was continued for 12 h at 50 °C.

[**Condition B**]: TMSCl (261 mg, 2.4 mmol, 1.2 equiv) was added to a mixture of imidazole (340 mg, 5 mmol, 2.5 equiv) and DMVC-OH (260 mg, 2.0 mmol, 1.0 equiv) in argon-purged dichloromethane (10 mL) at 0 °C. Stirring was continued for 12 h at room temperature (25 °C).

Synthesis of DMVC-OH by direct hydrolysis of DMVC-Br: the allyl bromide (DMVC-Br) obtained from DMVC through radical bromination is reactive toward nucleophilic substitution

reactions. Direct hydrolysis conditions were attempted; however, H_2O under neutral conditions revealed low conversion (Supplementary Table 2, entries 1,3; see also Supplementary Fig. 2 for TLC). Increasing the reaction temperature (60 °C) caused the decomposition of DMVC-Br, which is associated with the competitive electrophilicity of carbonate carbon of DMVC-Br (entries 2,4). The addition of NaOH did not facilitate DMVC-OH formation (entries 5,6). Thus, the hydroxymethyl (carbinol) moiety of DMVC-OH was introduced through a two-step sequence (Supplementary Fig. 3) using the readily hydrolysable formate ester intermediate with enhanced reaction yields[1]. The formate ester intermediate (DMVC-OCOH) was prepared from ally bromide (DMVC-Br) by using formic acid and triethylamine (TEA) in acetonitrile. Then, DMVC-OCOH was readily hydrolyzed to DMVC-OH (Supplementary Fig. 1).

Synthetic details of DMVC-Br[1]: DMVC (5.0 g, 1.0 equiv) was treated with *N*-bromosuccinimide (NBS, 8.2 g, 1.1 equiv) and azobisisobutyronitrile (AIBN, 98 μL) in 1,2-DCE (30 mL). The resulting mixture was stirred at 100 °C for 4 h. The title compound was yielded through flash chromatography (ethyl acetate/n-hexane, 7:1) and afforded the title product as a yellow oil (8.3 g, 98%); ^1H NMR (400 MHz, $CDC1_3$) δ 4.18 (s, 2H), 2.13 (s, 3H).

Synthetic details of DMVC-OH[1]: Triethylamine (18.1 mL, 3.0 equiv) was dropped into a solution of DMVC-Br (8.3 g, 1.0 equiv) and formic acid (4.9 mL, 3.0 equiv) in acetonitrile (80 mL) while keeping the temperature under 0 °C. The resulting mixture was stirred at room temperature for 2 h. After concentration of the reaction mixture, the residue was diluted with EtOAc. The organic phase was washed with water and brine and concentrated to produce DMVC-OCOH (5.0 g). This intermediate was then dissolved in methanol (75 mL), and 37% HCl (0.25 mL) was added. After stirring for 5 h at room temperature, the reaction mixture was concentrated. The title compound was yielded through flash chromatography (ethyl acetate/n-hexane, 1:1) as a

yellow oil (4.1 g, 73%); ^{1}H NMR (400 MHz, $CDCl_3$) δ 4.42 (s, 2H), 2.14 (s, 3H).

DFT calculations

DFT calculations were performed to investigate the LUMO energy levels and reaction mechanisms. Beck's three-parameter hybrid functional combined with the Lee-Yang-Parr correlation (B3LYP) functional was used for the exchange-correlation energy[2,3]. Spin-polarized calculations were employed, and a van der Waals correction was adopted by using the Tkatchenko-Scheffler method[4]. All relativistic core treatments and the double numerical plus polarization (DNP) version 4.4 basis set were used to describe the core electrons and the atomic orbital basis set, respectively. The convergence criteria for geometry optimization were 1.0×10^{-5} Ha for energy, 0.002 Ha/Å for force and 0.005 Å for displacement. The self-consistent field converged until the energy was less than 1.0×10^{-6} Ha. For the atomic charges on molecules, the Mulliken[5,6] charge analysis method was employed. The relative Gibbs free energy (ΔG) was calculated to investigate the reaction mechanism. The ΔG value was computed according to the equation S1:

$$\Delta G = \Delta H - T\Delta S \tag{S1}$$

where ΔH and ΔS are the changes in enthalpy and entropy from the reference state, respectively. ΔG can also be described as

$$\Delta G = H - H_{ref} - T\left(S - S_{ref}\right) = (U + PV) - \left(U_{ref} + P_{ref}V_{ref}\right) - T(S - S_{ref}) \tag{S2}$$

where U, P, V, and S are the molar internal energy, pressure, molar volume, and molar entropy, respectively. The subscript 'ref' indicates the reference state of each system. Equation S2 can be expressed with the vibrational, rotational, and translational motions, and ΔE (change in the electronic energy of the ground state) is calculated using the following equation:

$$\Delta G = \Delta E + (U' + PV) - \left(U'_{ref} + P_{ref}V_{ref}\right) - T(S - S_{ref}) \tag{S3}$$

where $U' = E_{vib} + E_{trans} + E_{rot}$ and $U'_{ref} = E_{ref,vib} + E_{ref,trans} + E_{ref,rot}$.

For the calculation of the transition state in the reaction mechanism, complete single linear synchronous transit (LST) and quadratic synchronous transit (QST) methods were used[7,8]. The root mean square (RMS) convergence force of the atoms was set to 0.01 Ha/Å. For the initial configurations of the polymerization reaction by the DMVC radical in Fig. 3d, Supplementary Figs. 11 and 16, Monte Carlo simulations were performed by using the Sorption program[9]. The rate of production steps was 1 × 10^6. The COMPASS II forcefield[10] was used to estimate the nonbonding interaction (*i.e.*, electrostatic and van der Waals interactions). For the atomic charges on molecules, Mulliken charges were adopted from the DFT calculations.

For the calculations of the adsorption energies of additives (DMVC-OCF_3, DMVC-OTMS, FEC, VC) on the Li (001) surface, the generalized gradient approximation (GGA) with the Perdew-Burke-Ernzerhof (PBE) functional[11] was used for the exchange-correlation energy. The orbital cutoff and smearing value were set to 5.1 Å and 0.005 Ha, respectively. The 4×5×1 *k*-point was used with Monkhorst-Pack grid[12].

Electrochemical evaluation

Two thousand thirty-two coin-type full cells (NCM811/Si-C, NCM622/Si-C, and NCM622/graphite) were cycled at a current density of C/5 (formation), C/2 (stabilization), and 1C (cyclability test, 1C = 2.7 mA cm^{-2}) between 4.3 V and 2.5 V using a computer-controlled battery measurement system (WonATech WBCS3000) in a constant-temperature chamber at 25 and 45 °C. To examine the effect of additives on the impedance of the full cells, AC impedance analysis was performed using an electrochemical workstation (Biologic, SP-300) in the frequency range of 10 mHz to 1 MHz at 25 °C. To examine the charge rate capability, full cells were cycled with different

charge current densities from 1 to 5C (1C, 2C, 3C, and 5C) with a fixed discharge current density of 1C between 4.3 V and 2.5 V. To evaluate the long-lasting, fast-charging capabilities, full cells were cycled at a charge/discharge current density of 1C/1C for 20 cycles and then cycled at a charge/discharge current density of 3C/1C for 100 cycles.

Characterization

The chemical structure of the SEI on the Si-C anode was identified with attenuated total reflection Fourier transform infrared spectroscopy (ATR-FTIR, 670-IR, Varian) and X-ray photoelectron spectroscopy (XPS, Scientific K-Alpha system, Thermo Scientific) with Al Kα radiation ($h\nu$ = 1486.6 eV) under ultrahigh vacuum. All XPS spectra were energy calibrated using the hydrocarbon peak at a binding energy of 284.8 eV. To investigate the transition metal content on the Si-C anode after cycling, the Si-C anodes were analyzed by inductively coupled plasma-optical emission spectrometry (ICP-OES, 700-ES, Varian). The Si-C anode particles (5 mg) retrieved from NCM811/Si-C after 400 cycles were treated with 16 ml of aqua regia in a PTFE container and heated at a constant temperature of 200 °C to reduce the volume of the mixture by 1 ml. A 1 wt% HNO_3 solution (49 ml) was then added to make up the mass. Finally, 50 ml of the solution was transferred to a conical tube for analysis.

Derivation of the Young's modulus of the Si nanolayer

The mechanical properties of battery electrodes have rarely been investigated. The surface contact stiffness of the SEI on a surface-modified Si nanowire was measured by Xu *et al.*[13], and the inhomogeneous SEI on a MnO anode was analyzed by Zhang *et al.*[14] using an atomic force microscopy (AFM) nanoindentation technique. To analyze the mechanical properties of the Si

nanolayer, NCM811/Si-C full cells were cycled and disassembled to retrieve the Si-C anode. Full cells were disassembled, and the retrieved Si-C anode was rinsed with dimethyl carbonate (DMC) in an argon-filled glove box. To locate the Si-C anode particles for conducting the AFM study, optical microscopy (LU Plan Fluor 50x BD, Nikon) was used. Si-C anode particles with a flat surface were chosen for analysis by the AFM nanoindentation method using the Sneddon *cone-on-flat* model[15]. Consequently, topographic images of the Si-C anode particles were obtained in tapping mode, and force-depth (FD) curves of 16 spots on the topographic image of the Si-C anode were obtained by the contact mode method using AFM (MultiMode V, Veeco) with a proper probe (PR-C13, Probes Inc., Korea). From the FD curves (Supplementary Fig. 40) of the Si nanolayer, the force applied to the probe ($F_{sneddon}$) and the depth ($S_0 - S$) were obtained according to equation 4, and the Young's modulus of the Si nanolayer ($E_{surface}$) was calculated. The tip half cone opening angle (α, Supplementary Fig. S39b) was 15°. The Poisson ratio ($v_{surface}$) was assumed to be 0.5, which is the conventional value for a rubber-like sample[16].

Supplementary Table 1. Optimization studies for the synthesis of DMVC derivatives

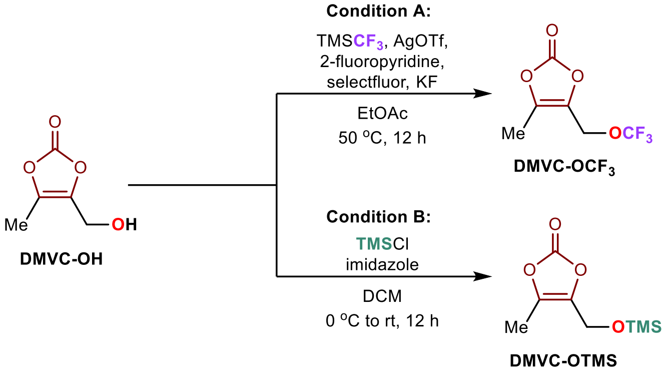

Entry	Changes from **Condition A**	Isolated yield (%): **DMVC-OCF$_3$**	Entry	Changes from **Condition B**	Isolated yield (%): **DMVC-OTMS**
A1	none	45	B1	none	87
A2	without TMSCF$_3$	0	B2	without TMSCl	0
A3	without AgOTf	0	B3	without imidazole	0
A4	AgF instead of AgOTf	0	B4	TMSBr instead of TMSCl	77
A5	CsF instead of KF	10	B5	TEA instead of imidazole	78
A6	room temperature	38	B6	50 °C	85
A7	6 h	25	B7	6 h	79

Supplementary Table 2. Attempted direct hydrolysis conditions of DMVC-Br

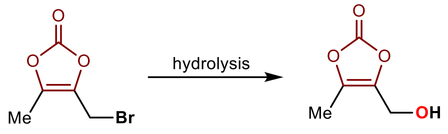

entry	attempted hydrolysis conditions	isolated yield (%): **DMVC-OH**
1	H_2O as a solvent at rt	<1%
2	H_2O as a solvent at 60 °C	2%
3	H_2O:MeCN = 1:1 as solvent at rt	<1%
4	H_2O:MeCN = 1:1 as solvent at 60 °C	<1%
5	NaOH (1 equiv) in H_2O: MeCN = 1:1 at rt	<1%
6	NaOH (1 equiv) in H_2O: MeCN = 1:1 at 60 °C	<1%

Supplementary Table 3. Cycling performance of full cells with Ni-rich cathodes and Si-containing anodes

Cathode	Mass loading of cathode (mg/cm^2)	Anode	Mass loading of anode (mg/cm^2)	Si composition in anode (%)	Operating voltage	Cycling performance		Ref.
						Ref.[a]	Exp.	
NCM811	13.5	Si-C	7.5	3	2.5 – 4.3 V	51.0% @ 1C, 400 cycles	81.5% @ 1C, 400 cycles	This work
	10	Si-graphite	2.2	12.5	2.7 – 4.4 V	55% @ 0.5C, 50 cycles	80% @ 0.5C, 50 cycles	17
	-	Si-graphite	5	3	2.8 – 4.25 V	-	85% @ 0.7C/1C, 50 cycles	18
	-	Si-graphite	-	5	2.5 – 4.2 V	-	83% @ 0.5C/0.2C, 250 cycles	19
	21	SiO-C	11.7	-	2.5 – 4.2 V	-	80% @ 0.5C, 200 cycles	20
	-	Si/C	-	16	2.5 – 4.2 V	-	84% @ 0.5C, 100 cycles	21
	4-5	SiO_x	1	-	3.0 – 4.2 V	25% @ 0.5C, 300 cycles	67.5% @ 0.5C, 300 cycles	22
NCM622	21.1	Si alloy-graphite	11.2	-	2.5 – 4.2 V	42.0% @ 1C/0.5C, 417 cycles	58.3% @ 1C/0.5C, 417 cycles	23
NCM523	10.26	Si-graphite	2.64	15	3.0 – 4.6 V	32% @ 0.1C, 100	82% @ 0.1C, 100	24

						cycles	cycles	
	3.4	Si-graphite	1.35	22.5	3.0 – 4.55 V	44% @ C/3, 100 cycles	71% @ C/3, 100 cycles	25

[a] Ref. represents the additive-free electrolytes.

Supplementary Table 4. Normalized proportion of chemical bonds of XPS C 1*s* spectra of Si-C anodes precycled with VC and VC + DMVC-OCF_3 + DMVC-OTMS

C 1*s*	284.8<br>C-C	286.0<br>C-O	287.0<br>C=O	288.5<br>O-C=O	290.3<br>Li_2CO_3	291.3<br>poly(VC)	292<br>CF_2
VC	23.7	44.1	20.5	5.6	2.7	3.5	0
VC + DMVC-OCF_3 + DMVC-OTMS	21.8	42.5	24.4	5.0	3.0	2.2	1.1

Supplementary Table 5. Normalized proportion of chemical bonds of XPS O 1*s* spectra of Si-C anodes precycled with VC and VC + DMVC-OCF_3 + DMVC-OTMS

O 1*s*	529.5<br>Metal-O	531.6<br>C=O	533<br>C-O-C
VC	36.8	49.0	14.2
VC + DMVC-OCF_3 + DMVC-OTMS	18.0	45.4	36.6

Supplementary Table 6. Normalized proportion of chemical bonds of F 1*s* XPS spectra of Si-C anodes precycled with VC and VC + DMVC-OCF_3 + DMVC-OTMS

F 1*s*	684.7<br>LiF	686.8<br>P-F
VC	61.9	38.1
VC + DMVC-OCF_3 + DMVC-OTMS	69.7	30.3

Supplementary Table 7. Normalized proportion of chemical bonds of O 1*s* XPS spectra of NCM811 cathodes precycled with VC and VC + DMVC-OCF_3 + DMVC-OTMS

O 1*s*	529.5<br>Metal-O	531.6<br>C=O	533<br>C-O-C
VC	12.3	41.3	46.4
VC + DMVC-OCF_3 + DMVC-OTMS	24.3	47.4	28.3

Supplementary Table 8. Normalized proportion of chemical bonds of XPS F 1*s* spectra of NCM811 cathodes precycled with VC and VC + DMVC-OCF_3 + DMVC-OTMS

F 1*s*	684.7<br>LiF	686.8<br>P-F	687.6<br>C-F
VC	38.8	26.3	34.9
VC + DMVC-OCF_3 + DMVC-OTMS	17.0	5.7	77.3

Supplementary Table 9. Normalized proportion of chemical bonds of XPS P 2*p* spectra of NCM811 cathodes precycled with VC and VC + DMVC-OCF_3 + DMVC-OTMS

P 2*p*	134.7<br>Li_xPOF_y	136.9<br>Li_xPF_y
VC	20.5	79.5
VC + DMVC-OCF_3 + DMVC-OTMS	59.3	40.7

Supplementary Table 10. ICP-OES analysis of Si-C anodes retrieved from NCM811/Si-C full cells with VC, FEC, VC + DMVC-OCF_3, and VC + DMVC-OCF_3 + DMVC-OTMS after 400 cycles

	Ni	Co	Mn
VC	225.0 ppm	53.5 ppm	35.6 ppm
FEC	96.6 ppm	21.7 ppm	14.9 ppm
VC + DMVC-OCF_3	55.2 ppm	19.8 ppm	13.9 ppm
VC + DMVC-OCF_3 + DMVC-OTMS	37.7 ppm	19.7 ppm	13.3 ppm

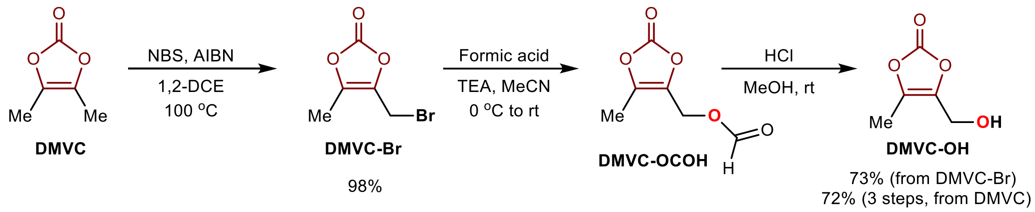

Supplementary Fig. 1. Synthesis of DMVC-OH through a formate ester intermediate.

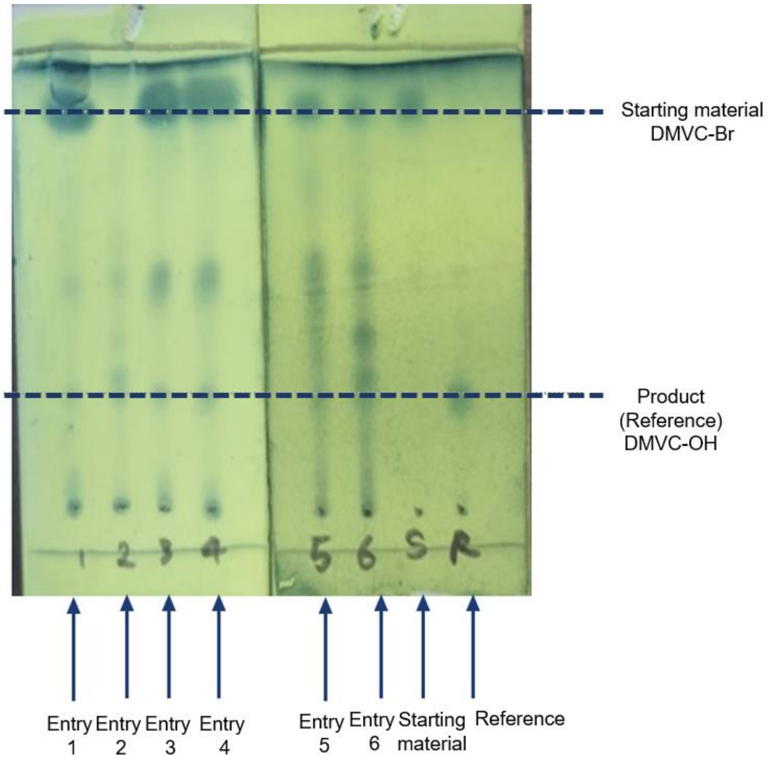

Supplementary Fig. 2. Attempted hydrolysis of DMVC-Br and TLC analysis.

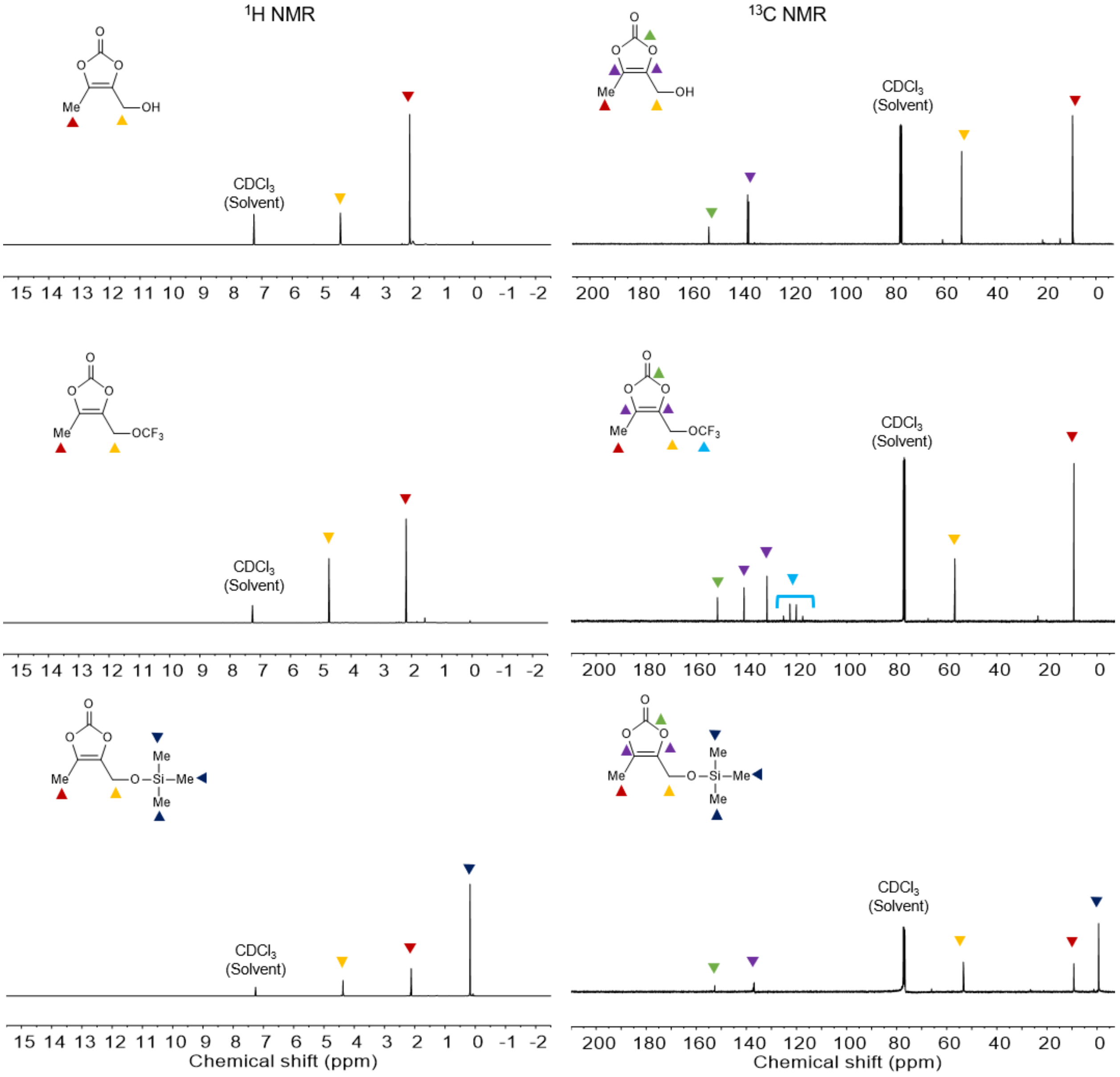

Supplementary Fig. 3. ^{1}H (400 MHz, $CDCl_3$) and ^{13}C NMR (101 MHz, $CDCl_3$) spectra of synthesized DMVC derivatives.

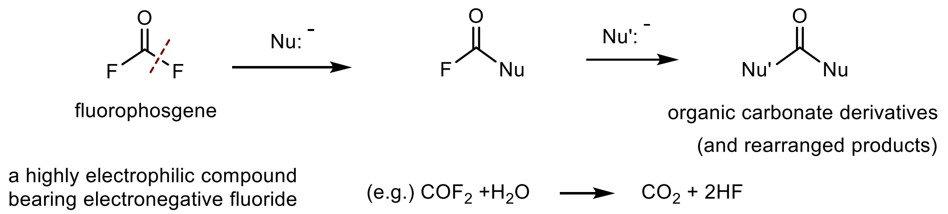

Supplementary Fig. 4. Conversion of COF_2 to secondary products.

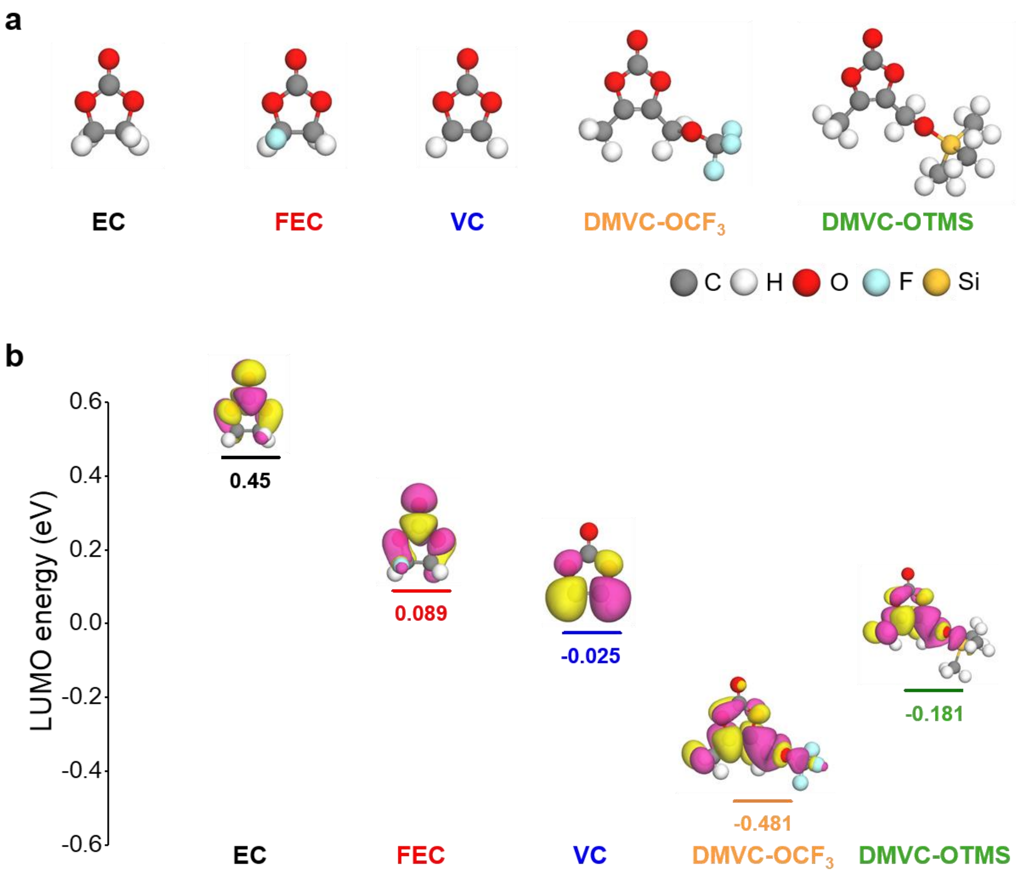

Supplementary Fig. 5. a, Molecular structures of EC and electrolyte additives (*i.e.*, FEC, VC, DMVC-OCF$_3$, and DMVC-OTMS). **b,** LUMO energy levels of EC, FEC, VC, DMVC-OCF$_3$, and DMVC-OTMS. The isovalue of the orbital is 0.02 e/Å^3.

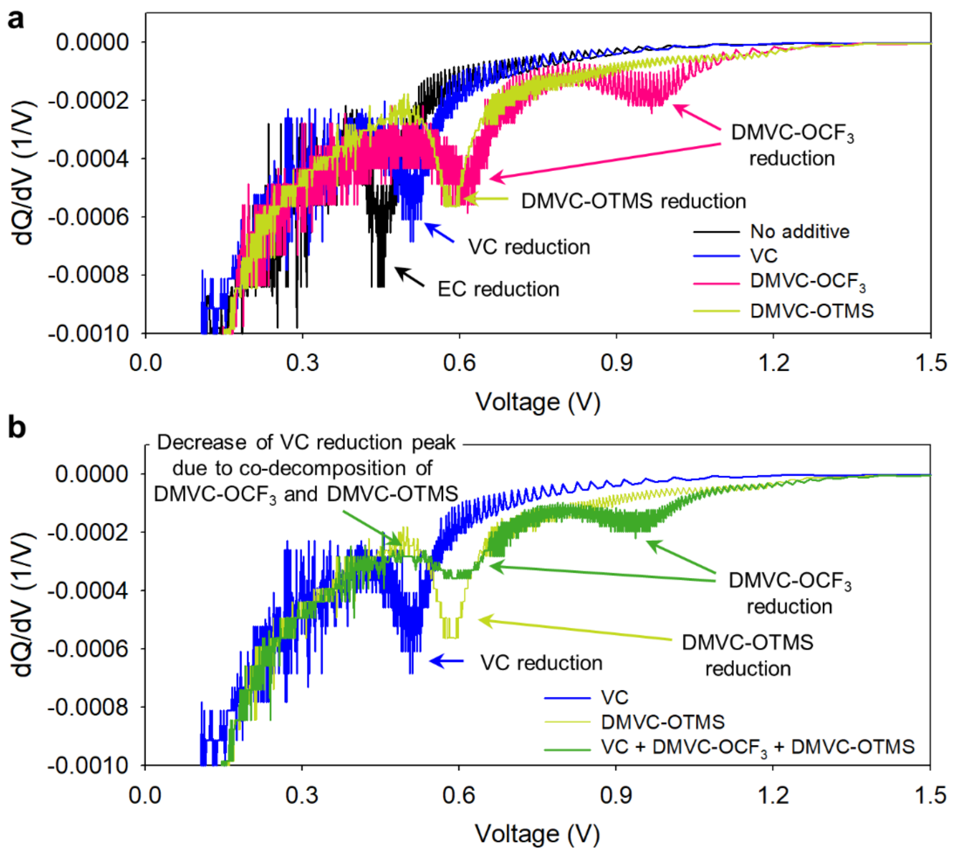

Supplementary Fig. 6. dQ/dV plots for the formation cycle of Si-C anode half cells with each synthesized additive (**a**) and a combination of additives (**b**). The disappearance of the EC reduction peak upon using DMVC-OCF_3 and DMVC-OTMS indicated that DMVC-OCF_3 and DMVC-OTMS modulated the interface structure of the Si-C anode (Supplementary Fig. 6b).

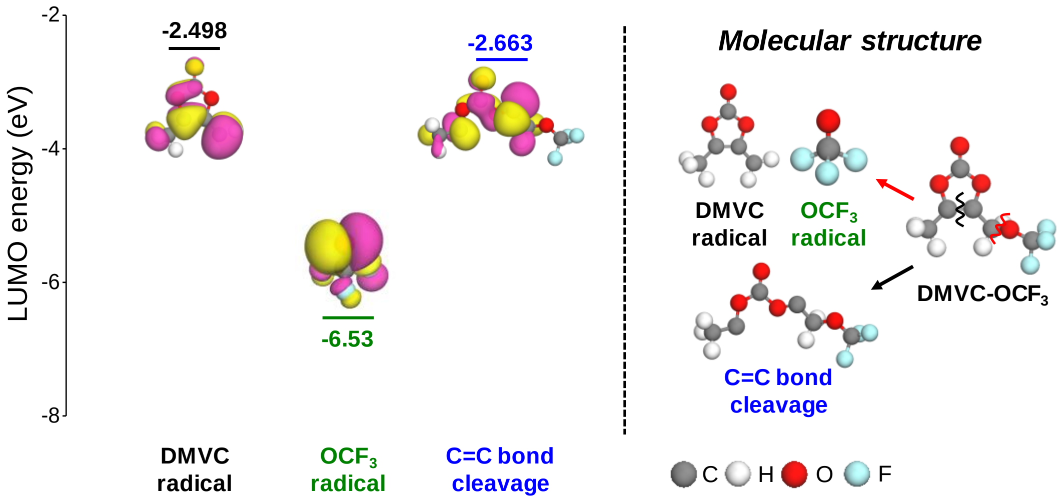

Supplementary Fig. 7. LUMO energy levels of the neutrally charged DMVC radical, OCF_3 radical, and C=C bond cleavage structures, which are possible products from the decomposition of DMVC-OCF_3. The isovalue of the orbital is 0.02 e/Å^3.

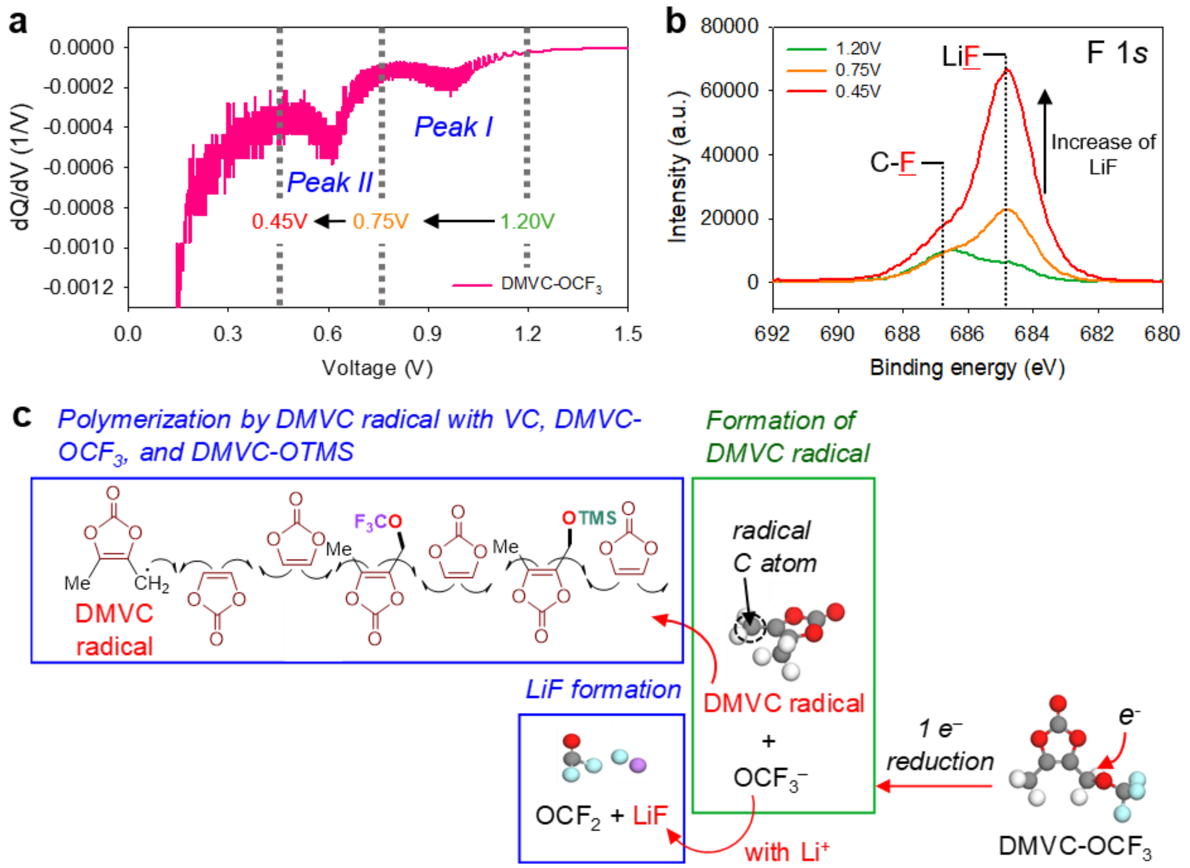

Supplementary Fig. 8. dQ/dV plot of the Li/Si-C half-cell with DMVC-OCF_3 (**a**), XPS F 1*s* spectra of Si-C anodes at different voltages during first lithiation (**b**), and reductive decomposition mechanisms of DMVC-CF_3 during charging of precycling at 25 °C (**c**).

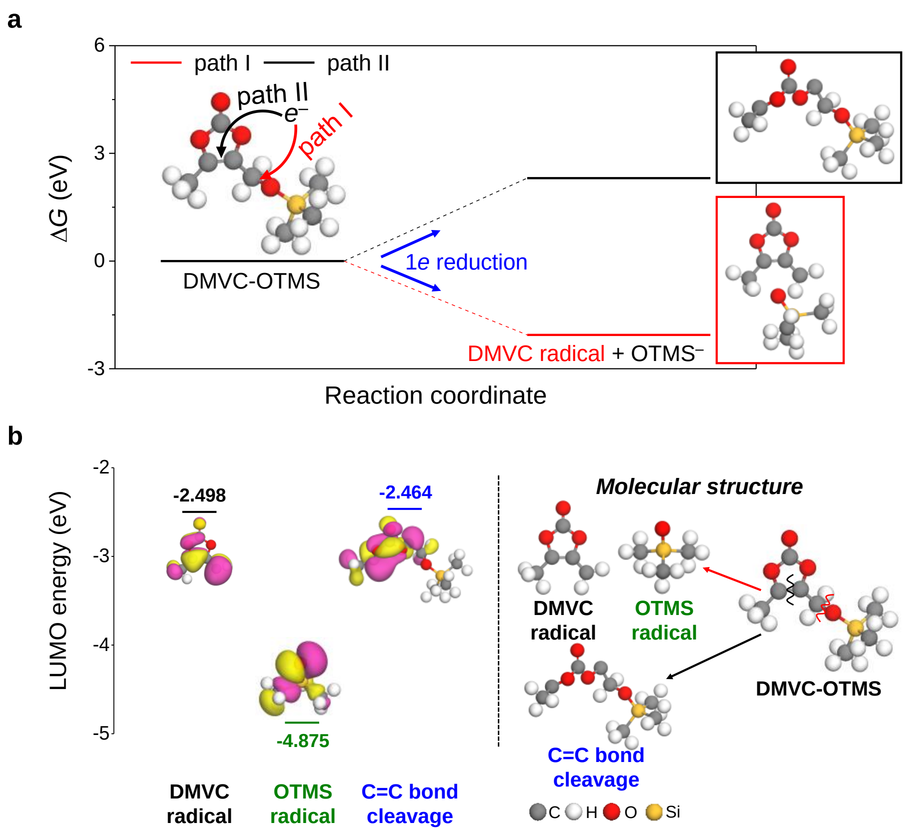

Supplementary Fig. 9. a, Reaction paths for decomposition of DMVC-OTMS by one-electron reduction. **b,** LUMO energy levels of neutrally charged DMVC radical, OTMS radical, and C=C bond cleavage structures, which are possible products from the decomposition of DMVC-OTMS. The isovalue of the orbital is 0.02 *e*/Å^3.

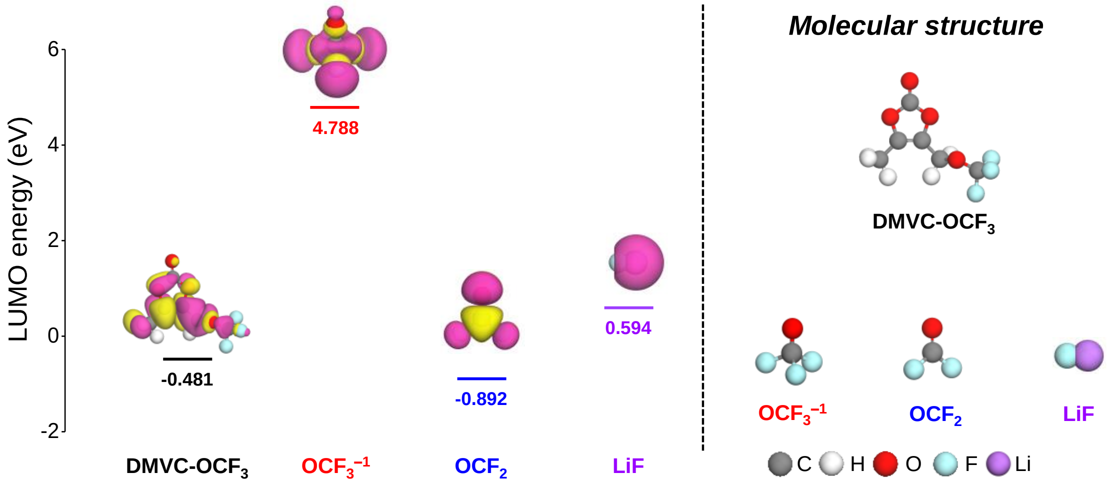

Supplementary Fig. 10. LUMO energy levels of DMVC-OCF_3, OCF_3 anion (OCF_3^{-1}), and OCF_2. The isovalue of the orbital is 0.02 e/Å^3.

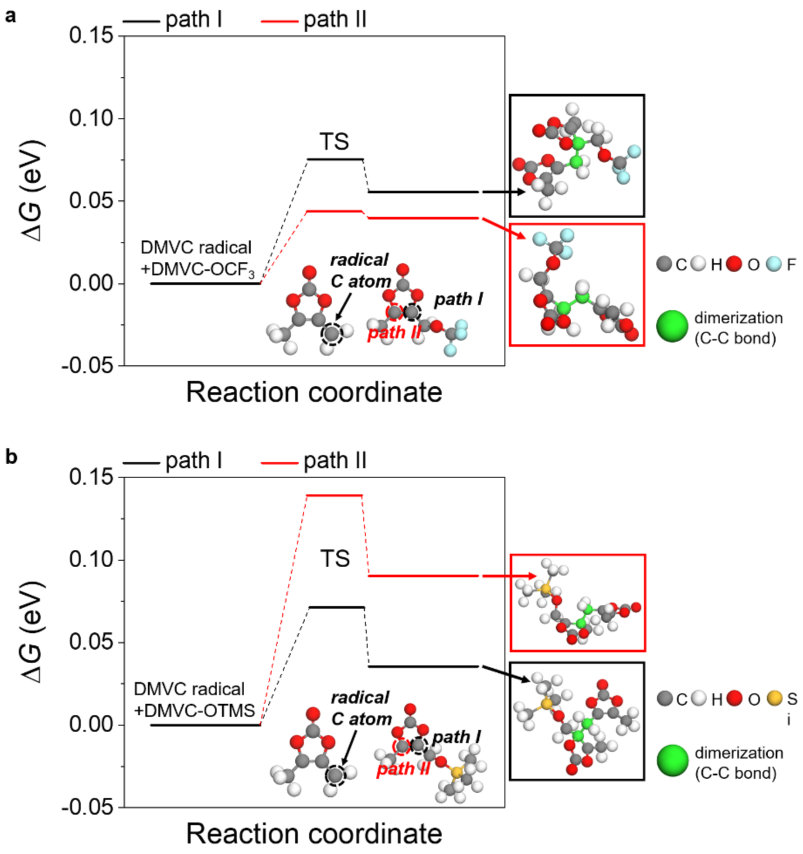

Supplementary Fig. 11. Reaction paths for the dimerization of DMVC radicals with DMVC-OCF_3 (**a**) or DMVC-OTMS (**b**). Relative Gibbs free energies (ΔG) are calculated at 1 atm and 298 K. The reactions occur by the radical C atom of the DMVC radical with the C atom of the vinyl group (path I and II) of DMVC-OCF_3 or DMVC-OTMS.

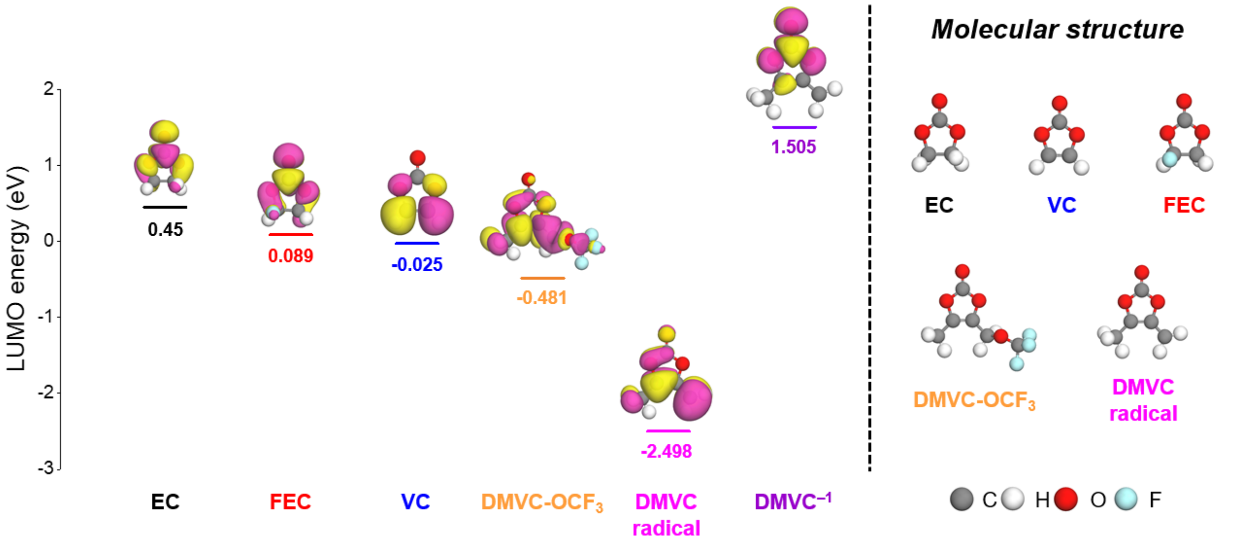

Supplementary Fig. 12. LUMO energy levels of EC, FEC, VC, DMVC-OCF$_3$, DMVC radical, and DMVC anion (DMVC^{-1}; one-electron reduction of DMVC radical). The orbital isovalue is 0.02 e/Å^3. Because DMVC radicals had a lower LUMO energy level than those of EC and the other electrolyte additives (FEC, VC, and DMVC-OCF$_3$), the decomposition reactions of DMVC radicals under the neutral conditions and in one-electron reduced states were investigated (Supplementary Figs. 13 and 14). Note that the condition of two-electron reduction was not considered because the DMVC anion can barely be reduced prior to the EC and the other electrolyte additive due to its high LUMO energy level.

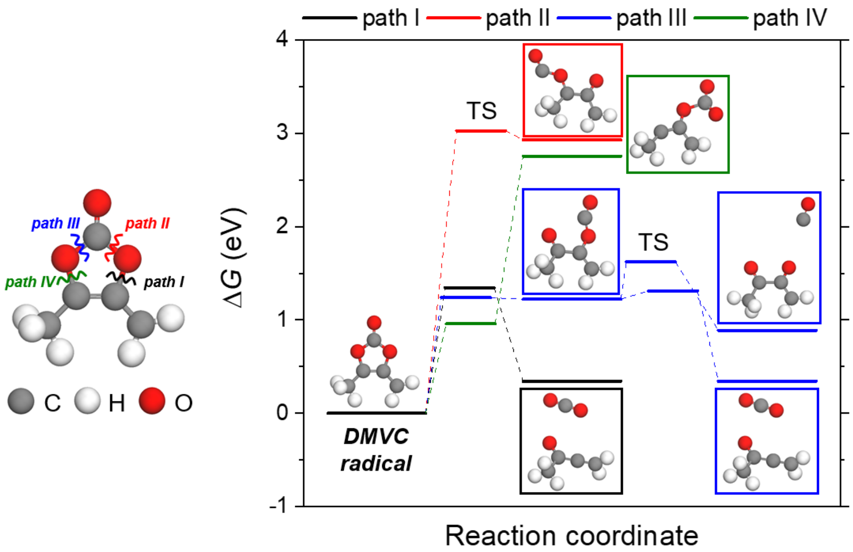

Supplementary Fig. 13. Reaction paths for the decomposition of DMVC radicals in the neutral charge state. Relative Gibbs free energies (ΔG) were calculated at 1 atm and 298 K.

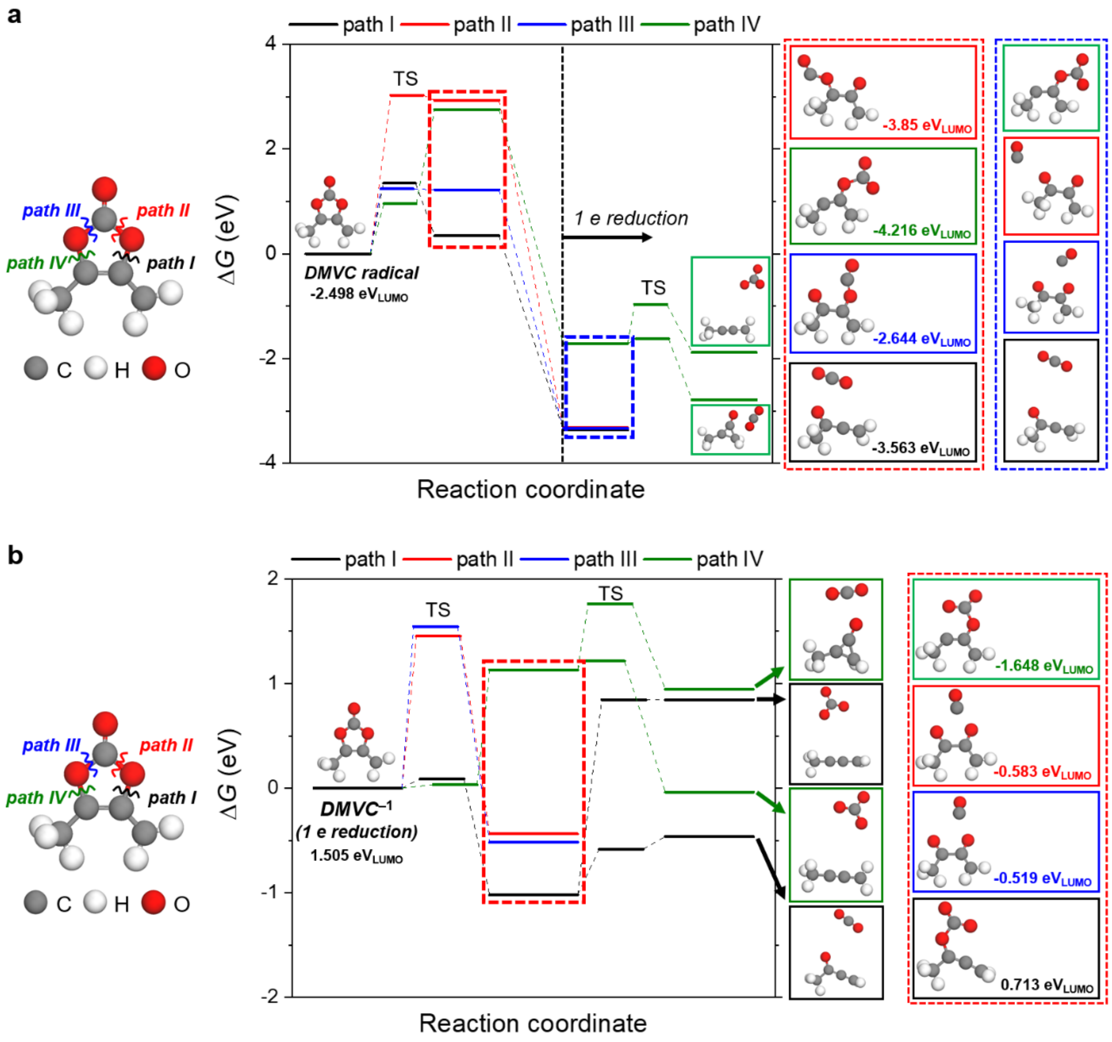

Supplementary Fig. 14. Reaction paths for the decomposition of DMVC radicals in the neutral charge state and successive one-electron (1 *e*) reduction (**a**) and 1 *e* reduction (**b**). Relative Gibbs free energies (Δ*G*) are calculated at 1 atm and 298 K. In each figure, LUMO energy levels are presented to investigate the possibility of an additional reduction of decomposed species. In the red dashed box of Fig. S14a, because all decomposed species from path I to IV had lower LUMO energy levels than DMVC radicals, 1 *e* reduction reactions of decomposed species were considered. In the red dashed box of Fig. S14b, because all decomposed species from path I to IV had higher LUMO energy levels than the DMVC radical (-2.498 eV_{LUMO}), even though the DMVC anion had a much higher LUMO energy level (1.505 eV_{LUMO}), an additional reduction (*i.e.*, 2 *e* reduction) was not considered.

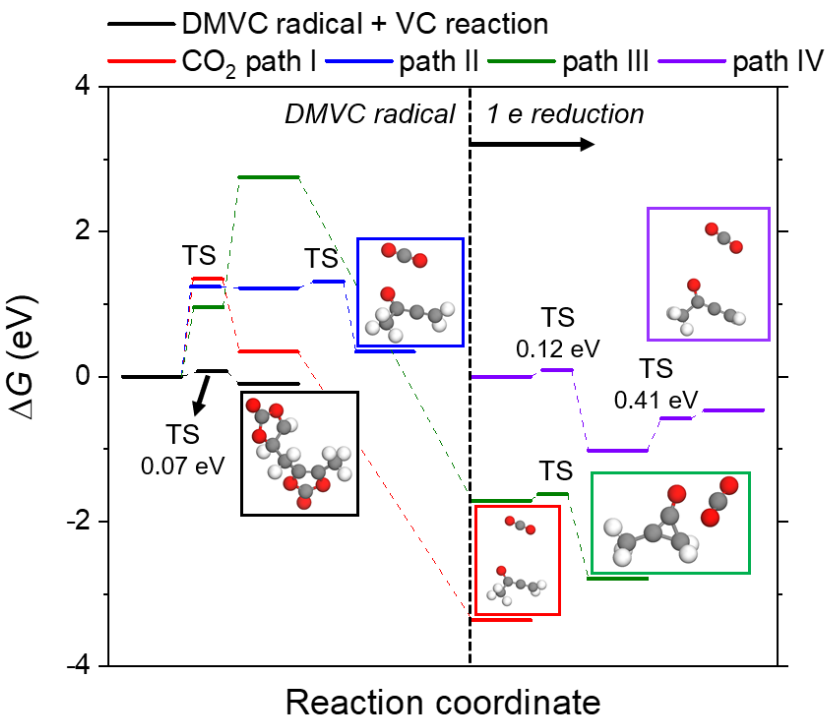

Supplementary Fig. 15. Reaction paths for dimerization by the DMVC radical with VC (Fig. 3d) and CO_2 generation from the decomposition of the DMVC radical (Supplementary Figs. 13 and 14) to compare these two types of reactions. Relative Gibbs free energies (ΔG) were calculated at 1 atm and 298 K. Gray, white, and red spheres denote C, H, and O atoms, respectively.

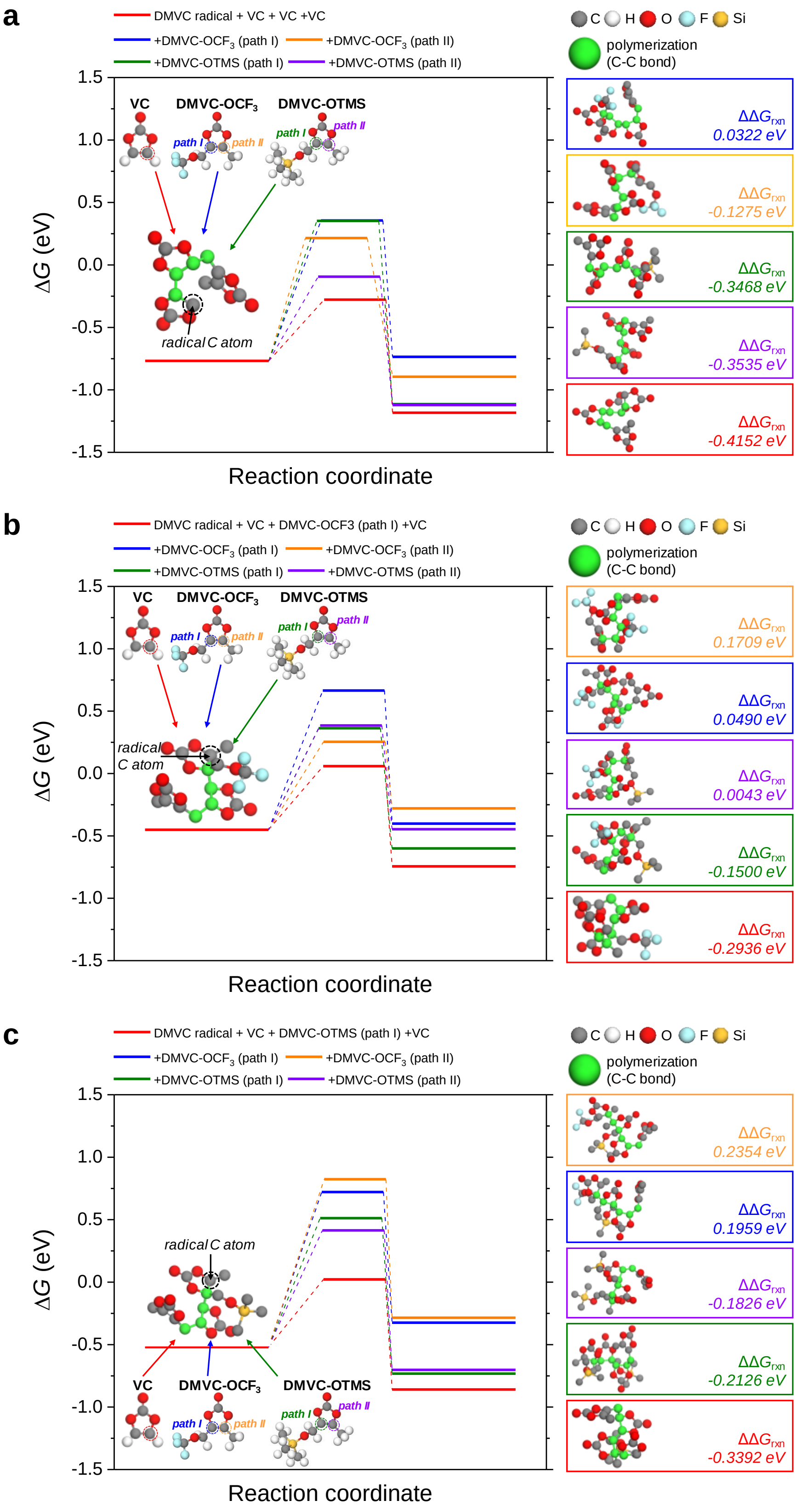
a
DMVC radical + VC + VC +VC
+DMVC-OCF3 (path I)
+DMVC-OCF3 (path II)
+DMVC-OTMS (path I)
+DMVC-OTMS (path II)
C H O F Si
polymerization (C-C bond)
VC
DMVC-OCF3
DMVC-OTMS
path I
path II
radical C atom
ΔG (eV)
Reaction coordinate
ΔΔG rxn 0.0322 eV
ΔΔG rxn -0.1275 eV
ΔΔG rxn -0.3468 eV
ΔΔG rxn -0.3535 eV
ΔΔG rxn -0.4152 eV
b
DMVC radical + VC + DMVC-OCF3 (path I) +VC
+DMVC-OCF3 (path I)
+DMVC-OCF3 (path II)
+DMVC-OTMS (path I)
+DMVC-OTMS (path II)
radical C atom
ΔG (eV)
Reaction coordinate
ΔΔG rxn 0.1709 eV
ΔΔG rxn 0.0490 eV
ΔΔG rxn 0.0043 eV
ΔΔG rxn -0.1500 eV
ΔΔG rxn -0.2936 eV
c
DMVC radical + VC + DMVC-OTMS (path I) +VC
+DMVC-OCF3 (path I)
+DMVC-OCF3 (path II)
+DMVC-OTMS (path I)
+DMVC-OTMS (path II)
radical C atom
ΔG (eV)
Reaction coordinate
ΔΔG rxn 0.2354 eV
ΔΔG rxn 0.1959 eV
ΔΔG rxn -0.1826 eV
ΔΔG rxn -0.2126 eV
ΔΔG rxn -0.3392 eV

Supplementary Fig. 16. Reaction paths for the tetramerization of DMVC radical + VC + VC (**a**), DMVC radical + VC + DMVC-OCF_3 (**b**), and DMVC radical + VC + DMVC-OTMS (omission of H atoms) (**c**) with VC, DMVC-OCF_3, and DMVC-OTMS. For the cases of DMVC radical + VC + DMVC-OCF_3 and DMVC radical + VC + DMVC-OTMS, the molecular configurations of path I in Fig. 3d were considered due to their lower energy barrier for an exothermic reaction compared to the results of path II. Relative Gibbs free energies (ΔG) were calculated at 1 atm and 298 K. $\Delta\Delta G_{rxn}$ indicates the difference in ΔGs between the product and reactant, which represents the heat of reaction. For the molecular structures in colored boxes, the hydrogen atoms are omitted for clarity.

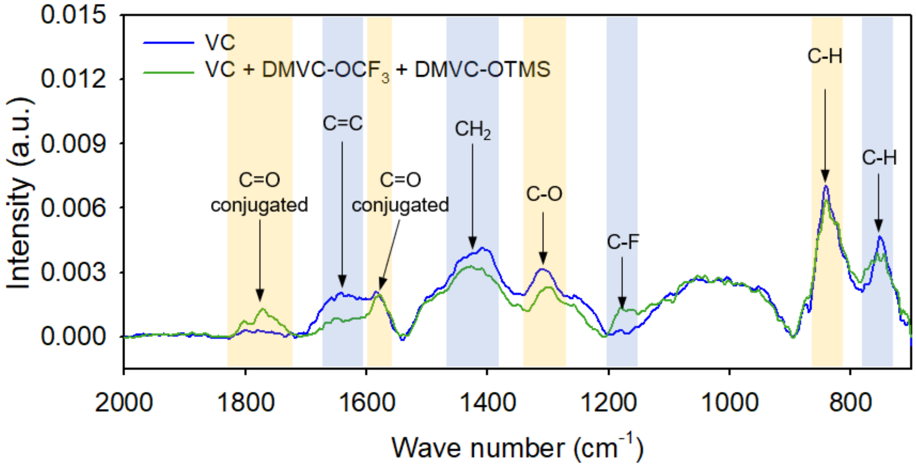

Supplementary Fig. 17. FT-IR spectra of the Si-C anode after precycling of NCM811/Si-C full cells.

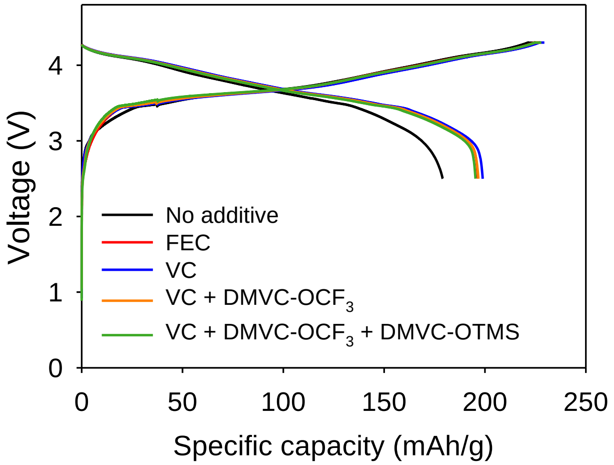

	Charge capacity (mAh/g)	Discharge capacity (mAh/g)	Initial Coulombic efficiency (%)
No additive	224.4	179.0	79.8
FEC	227.2	196.5	86.5
VC	229.4	198.9	86.7
VC + DMVC-OCF_3	227.8	196.7	86.3
VC + DMVC-OCF_3 + DMVC-OTMS	227.9	195.3	85.7

Supplementary Fig. 18. Voltage profiles of NCM811/Si-C full cells with commercial and synthesized additives during precycling at C/5.

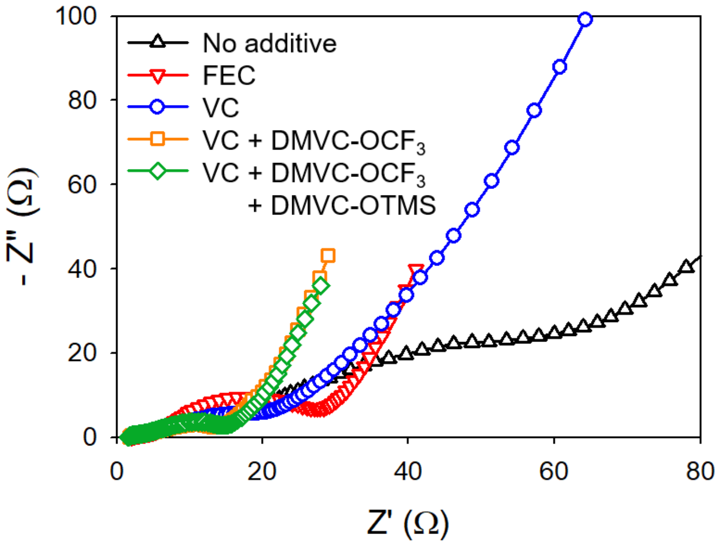

	R_{SEI} (Ω)	R_{CT} (Ω)	R_i (Ω)
No additive	16.2	87.8	104.0
FEC	3.9	26.1	30.0
VC	4.8	26.2	31.0
VC + DMVC-OCF_3	2.5	13.2	15.7
VC + DMVC-OCF_3 + DMVC-OTMS	2.6	14.9	17.5

Supplementary Fig. 19. Nyquist plots of NCM811/Si-C full cells after 400 cycles and the calculated R_{SEI} (SEI resistance), R_{CT} (charge transfer resistance), and R_i (interfacial resistance) values using an equivalent circuit model.

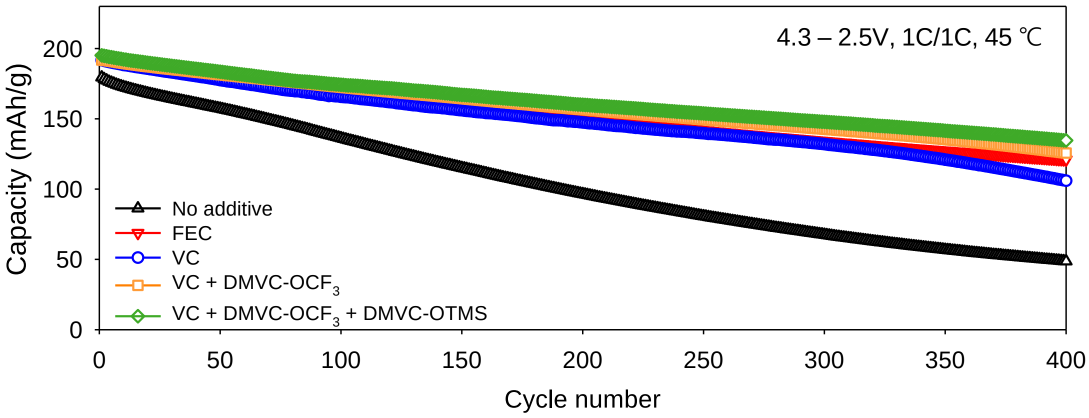

Supplementary Fig. 20. Cycle performance of NCM811/Si-C full cells at a 1C rate and 45 °C.

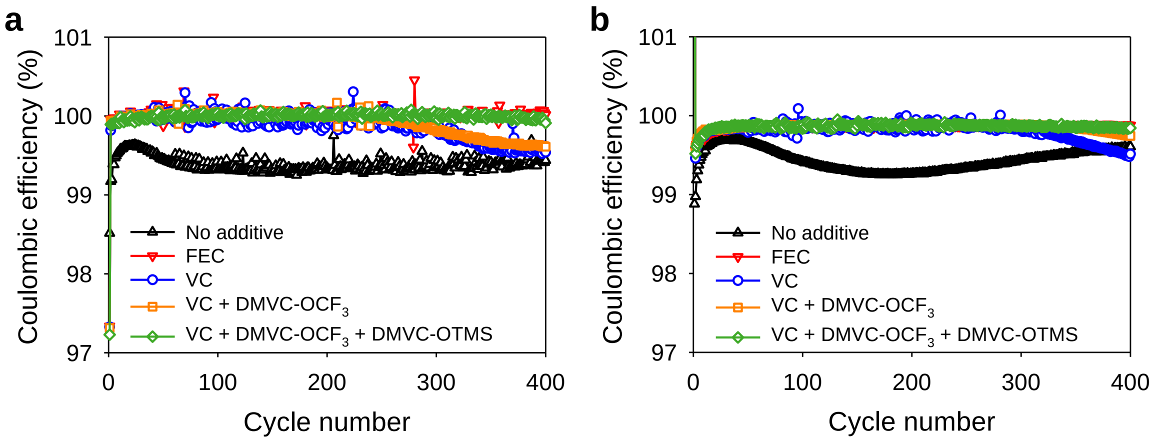

Supplementary Fig. 21. Cyclability test for the Coulombic efficiency of NCM811/Si-C full cells at a 1C rate and 25 °C (**a**) and 45 °C (**b**).

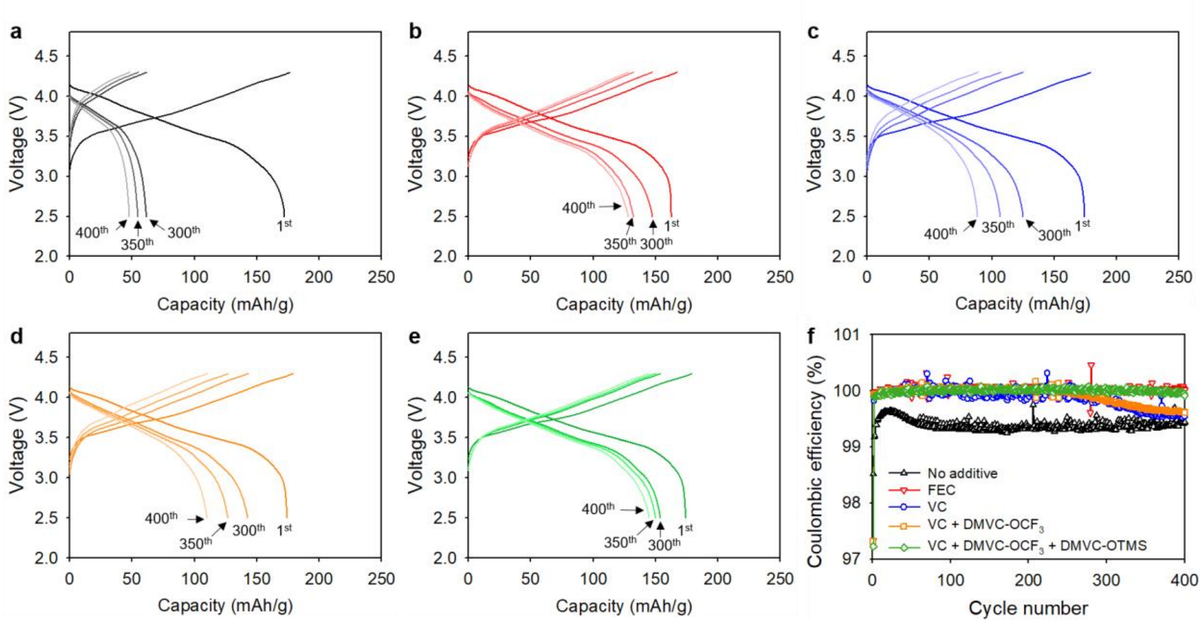

Supplementary Fig. 22. Voltage profiles of NCM811/Si-C full cells at a 1C rate and 25 °C with the no additive electrolyte (**a**), FEC containing electrolyte (**b**), VC containing electrolyte (**c**), VC + DMVC-OCF_3 containing electrolyte (**d**), and VC + DMVC-OCF_3 + DMVC-OTMS containing electrolyte (**e**) at the 1st, 300th, 350th, and 400th cycles. Cyclability test for the Coulombic efficiency of NCM811/Si-C full cells at a 1C rate and 25 °C (**f**).

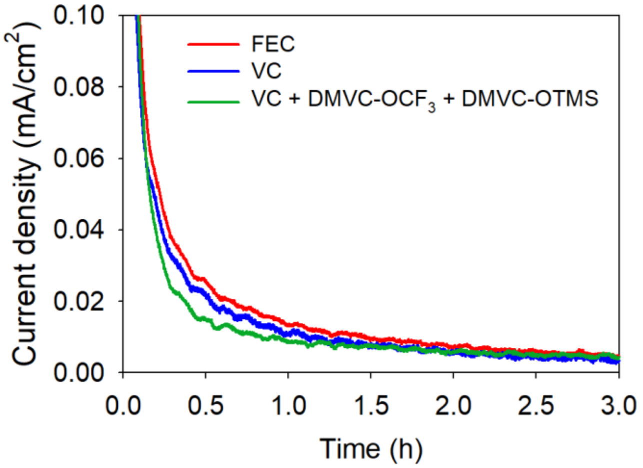

Supplementary Fig. 23. Leakage current density of Li/NCM811 half-cells with different electrolytes at a constant voltage of 4.35 V vs. Li/Li^+.

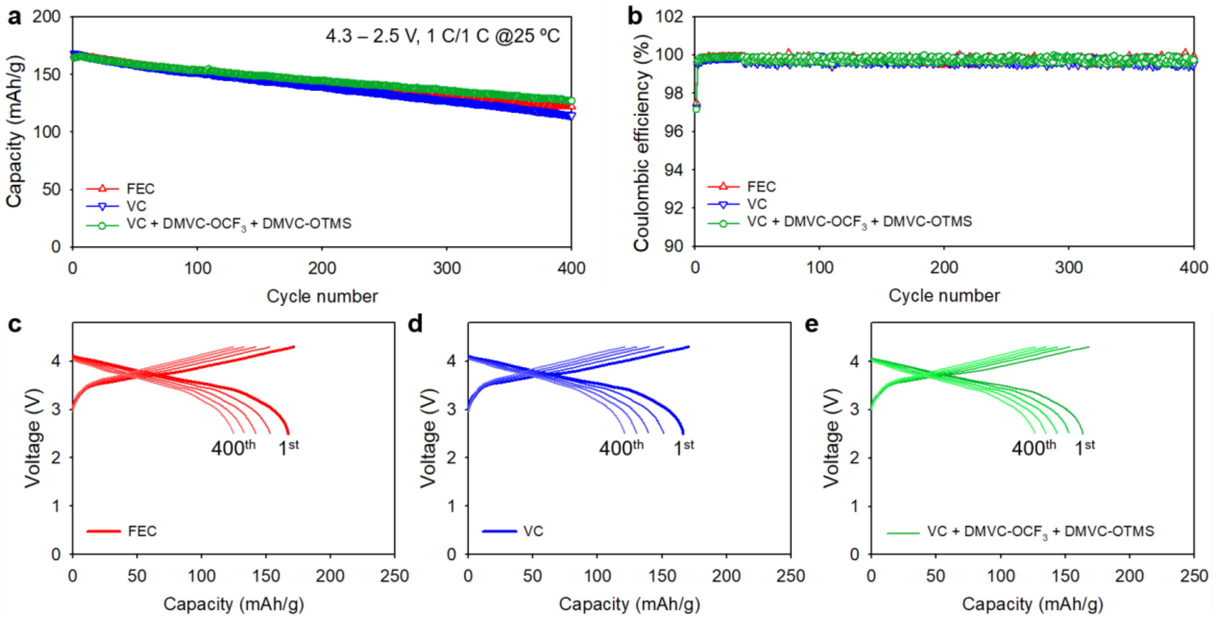

Supplementary Fig. 24. Cycle performance (**a**) and Coulombic efficiency (**b**) of Si-C anodes based on SNG with 7 wt% Si (520 mAh g^{-1}) coupled with NCM811 cathodes at a 1C rate and 25 °C. Voltage profiles of Si-C anodes based on SNG with 7 wt% Si (520 mAh g^{-1}) coupled with NCM811 cathodes at 1C and 25 °C with the FEC containing electrolyte (**c**), VC containing electrolyte (**d**), and VC + DMVC-OCF_3 + DMVC-OTMS containing electrolyte (**e**) at the 1st, 100th, 200th, 300th, and 400th cycles.

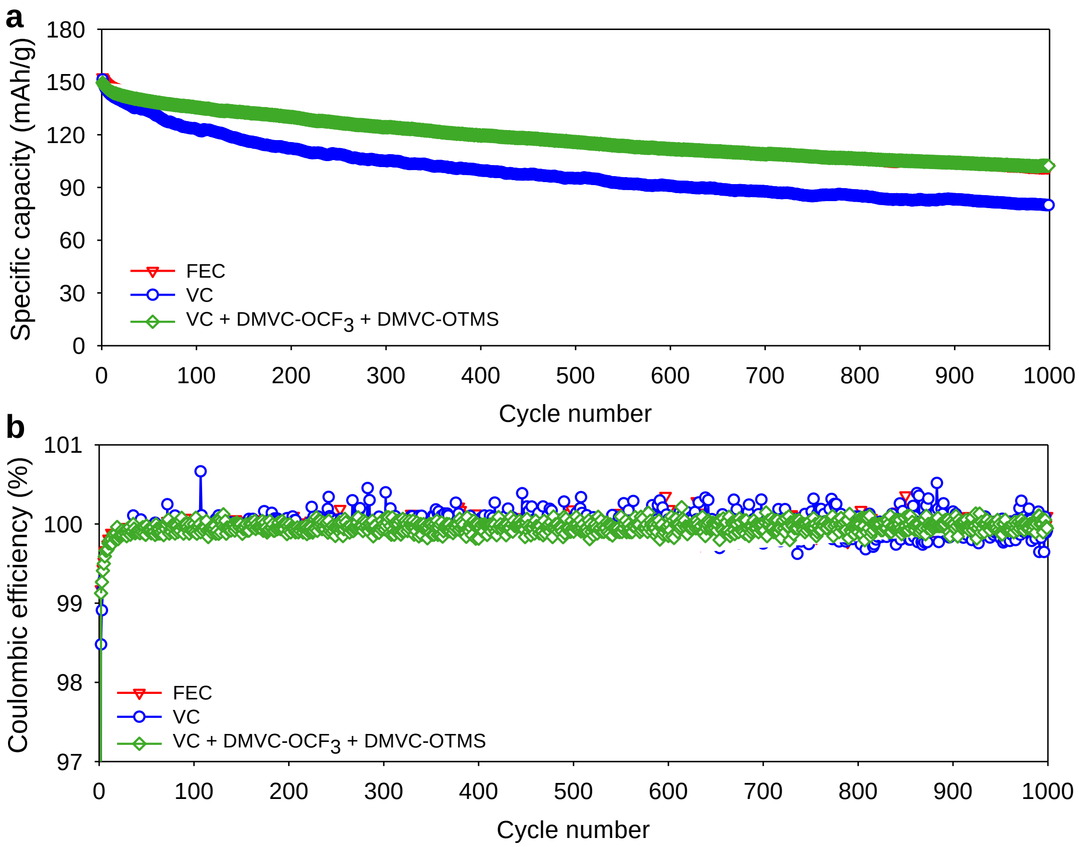

Supplementary Fig. 25. Long-term cycle performance (**a**) and Coulombic efficiency (**b**) of NCM811/Si-C full cells at a 1C rate and 25 °C with a depth of discharge (DOD) of 80%.

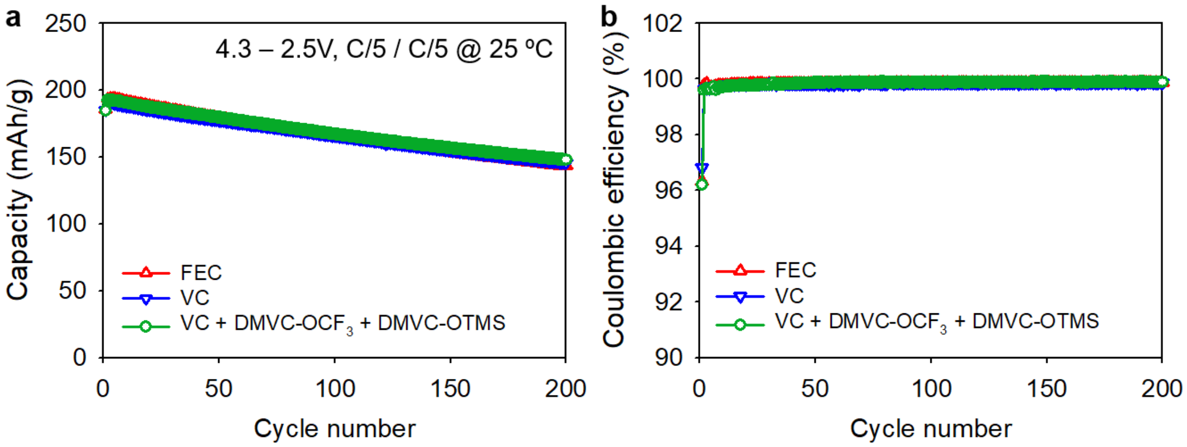

Supplementary Fig. 26. Cycle performance of the NCM811/Si-C full cells with different electrolytes at a C/5 rate and 25 °C.

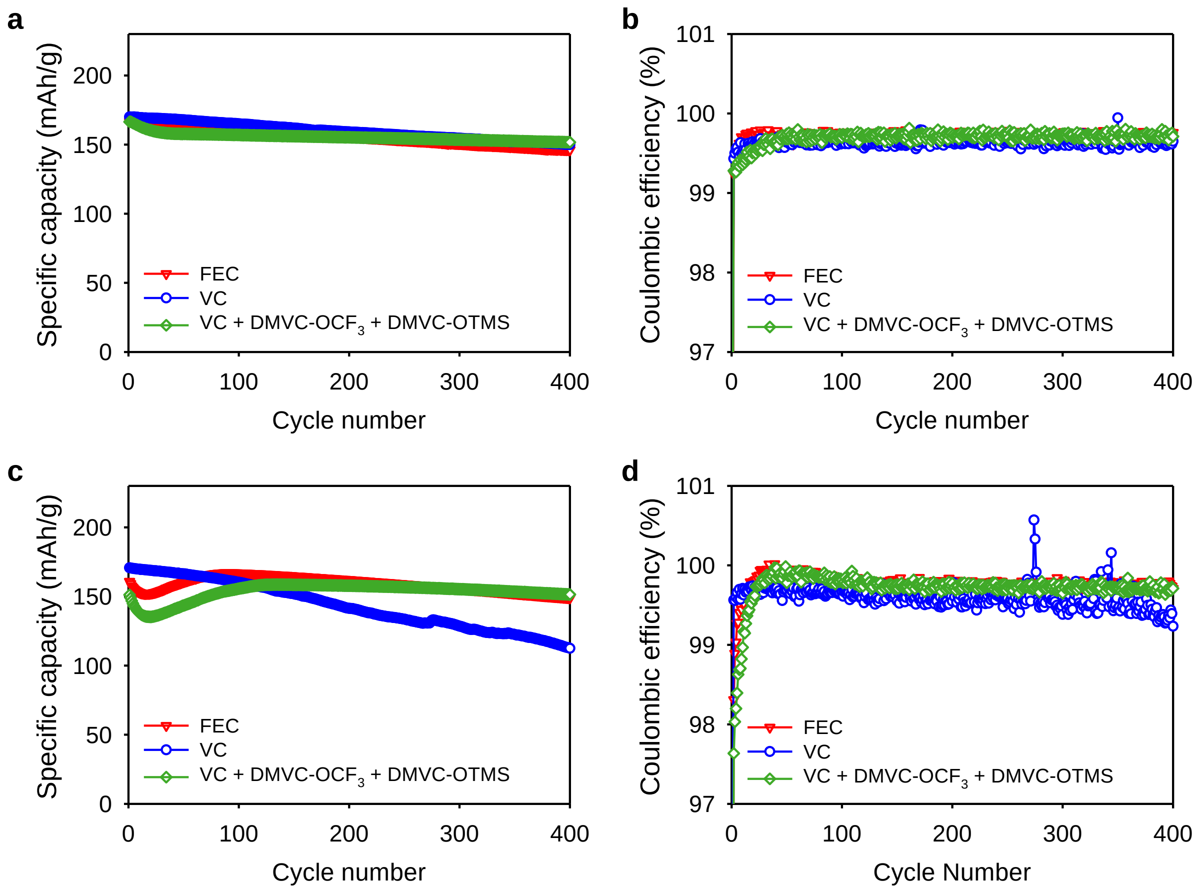

Supplementary Fig. 27. Cycle performance (**a**) and Coulombic efficiency (**b**) of NCM622/graphite full cells at a 1C rate and 25 °C. Cycle performance (**c**) and Coulombic efficiency (**d**) of NCM622/Si-C full cells at a 1C rate and 25 °C.

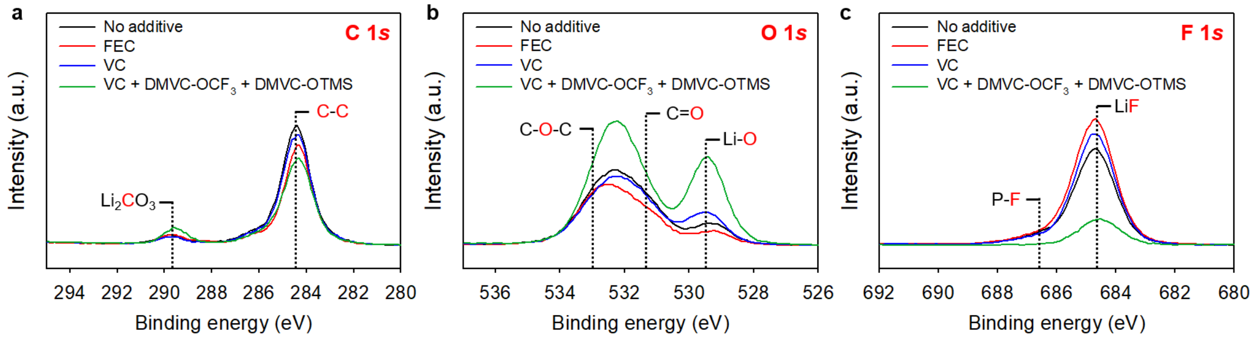

Supplementary Fig. 28. C 1*s* (**a**), O 1*s* (**b**), and F 1*s* XPS spectra (**c**) of Li metal electrodes retrieved from Si-C anode half-cells aged in different electrolytes for 20 h.

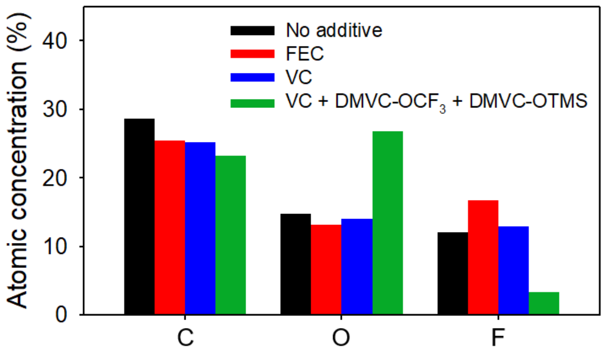

Supplementary Fig. 29. Composition of SEI on Li metal electrodes retrieved from Si-C anode half-cells aged in different electrolytes at 25 °C for 20 h.

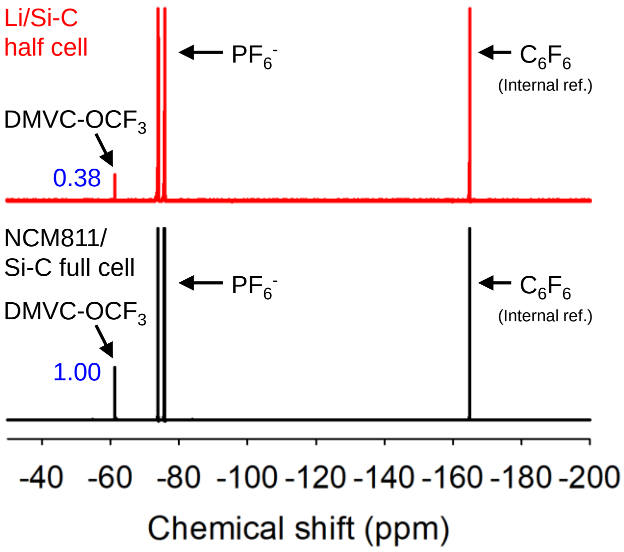

Supplementary Fig. 30. ^{19}F NMR spectra of a VC + DMVC-OCF_3 + DMVC-OTMS electrolyte stored in a Li/Si-C half-cell and NCM811/Si-C full cell for 20 h. Peak areas corresponding to –OCF_3 of DMVC-OCF_3 were calculated relative to the peak area of an internal standard (C_6F_6).

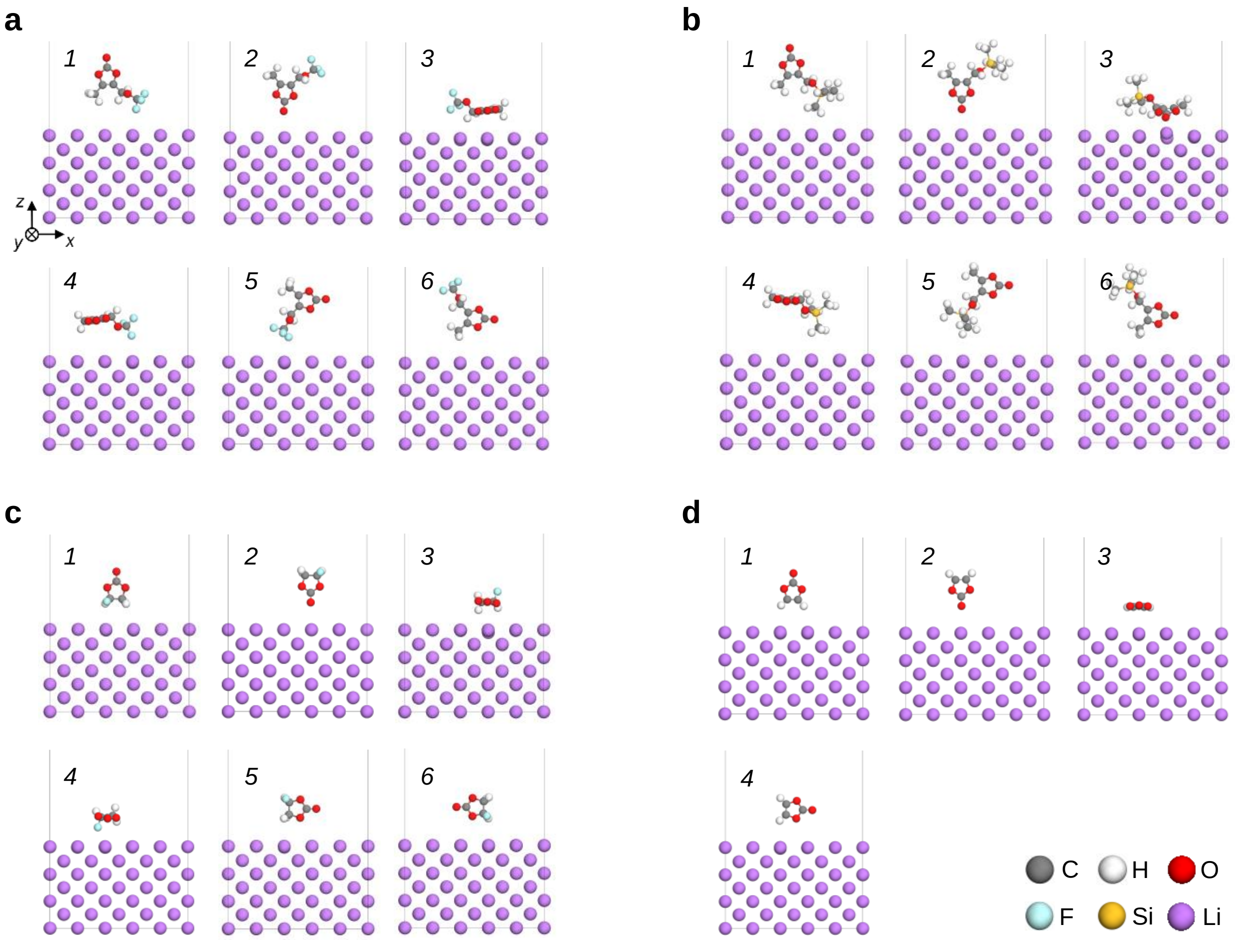

Supplementary Fig. 31. Model systems of DMVC-OCF_3 (**a**), DMVC-OTMS (**b**), FEC (**c**), and VC (**d**) on the (001) surface of Li metal. Note that various types of additive configuration were considered. The surface of Li metal consists of seven layers, which are the optimized number of layers in the (001) surface of Li metal[26], and the 5 × 4 supercell along the *x*- and *y*-axis. The two bottom layers are constrained. The vacuum spacing of model systems exceeds 20 Å.

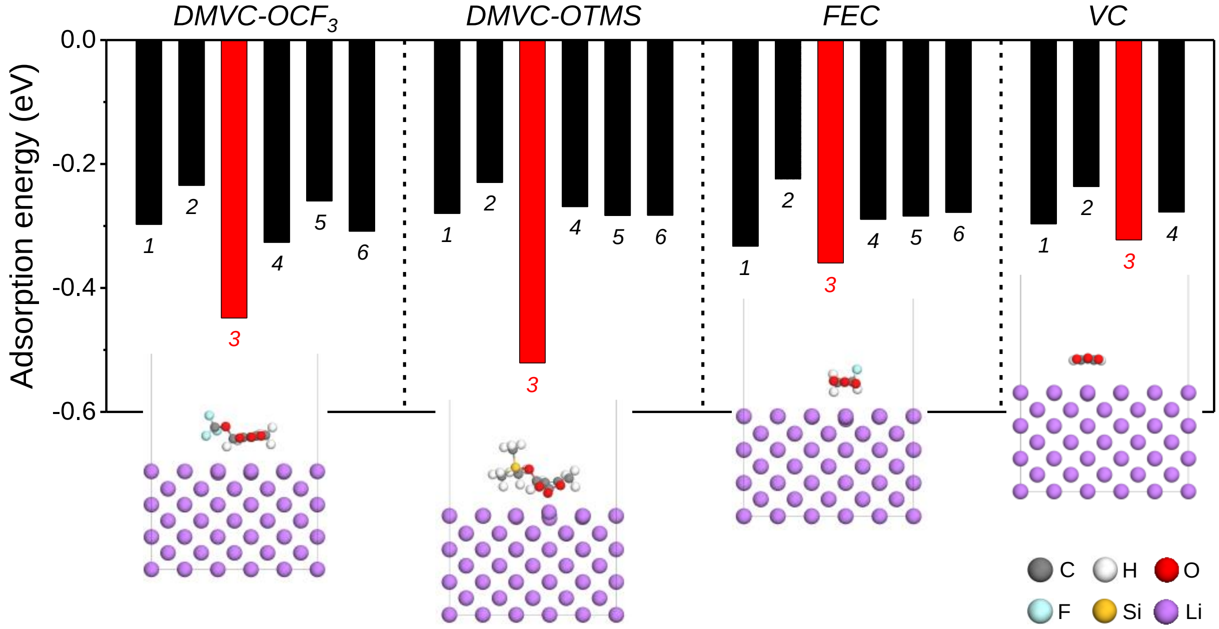

Supplementary Fig. 32. Adsorption energies of various configurations of DMVC-OCF$_3$, DMVC-OTMS, FEC or VC on the Li (001) surface. The number labels below the bars indicate each adsorption configuration presented in Supplementary Fig. 31.

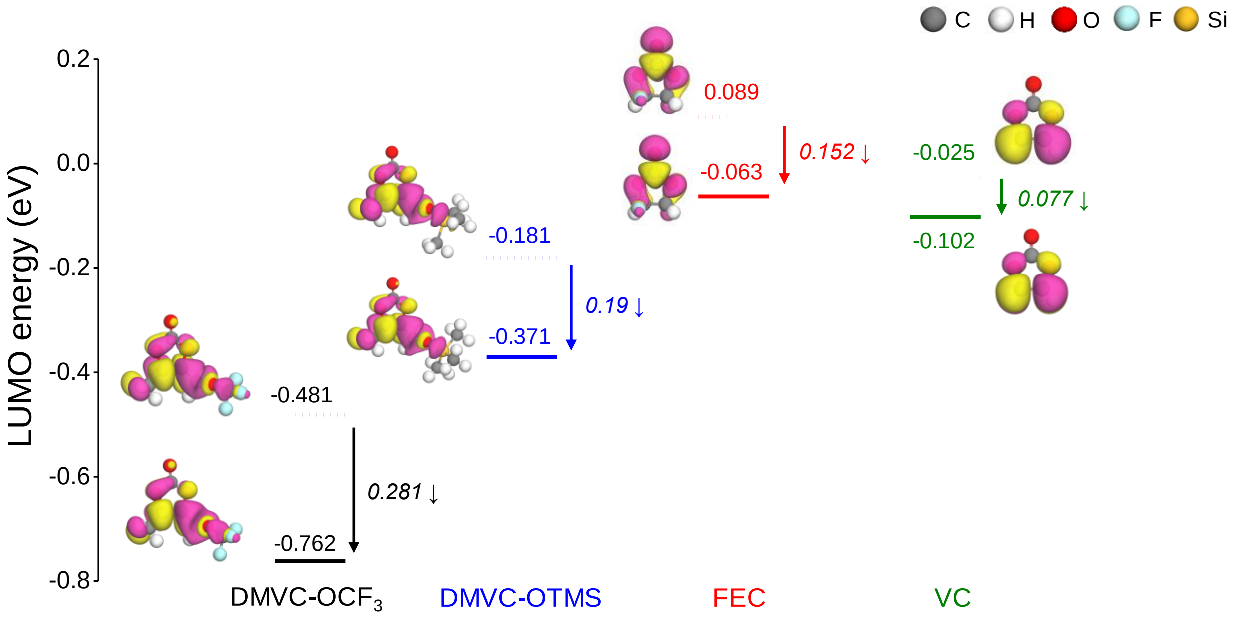

Supplementary Fig. 33. Changes in the LUMO energy levels of additives with adsorption on the Li metal surface. The dotted and solid lines indicate the LUMO energy levels of additives before and after adsorption on the Li metal surface, respectively. The isovalue of the orbital is 0.02 e/Å^3.

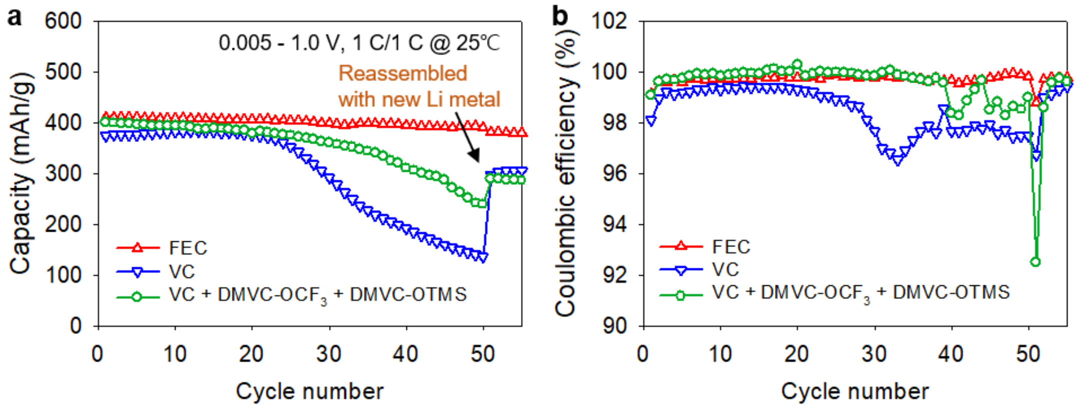

Supplementary Fig. 34. Cycle performance (**a**) and Coulombic efficiency (**b**) of Li/Si-C half-cells at 1C and 25 °C after five formation cycles at a C/5 rate.

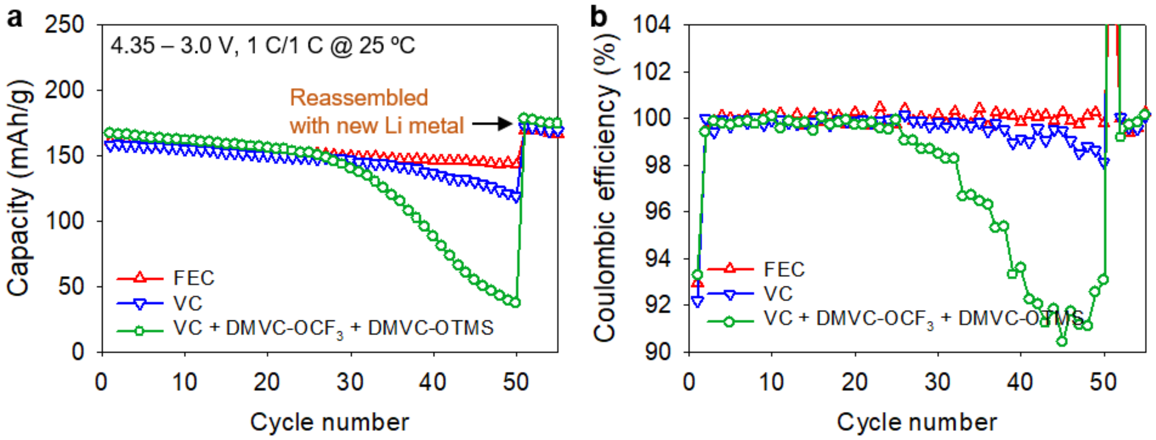

Supplementary Fig. 35. Cycle performance (**a**) and Coulombic efficiency (**b**) of Li/NCM811 half-cells at 1C and 25 °C after five formation cycles at a C/5 rate.

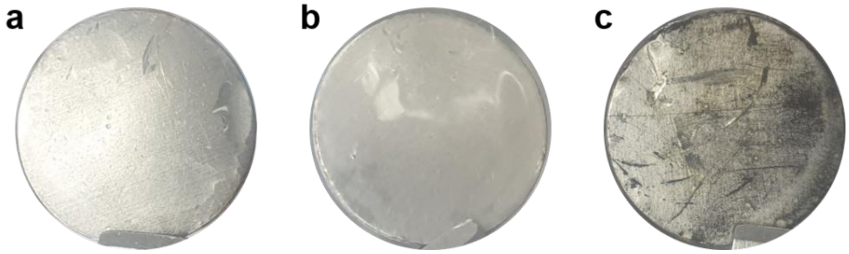

Supplementary Fig. 36. Li metal electrodes stored with FEC **(a)**, VC **(b)**, and DMVC-OCF_3 **(c)** after 1 day at 25 °C.

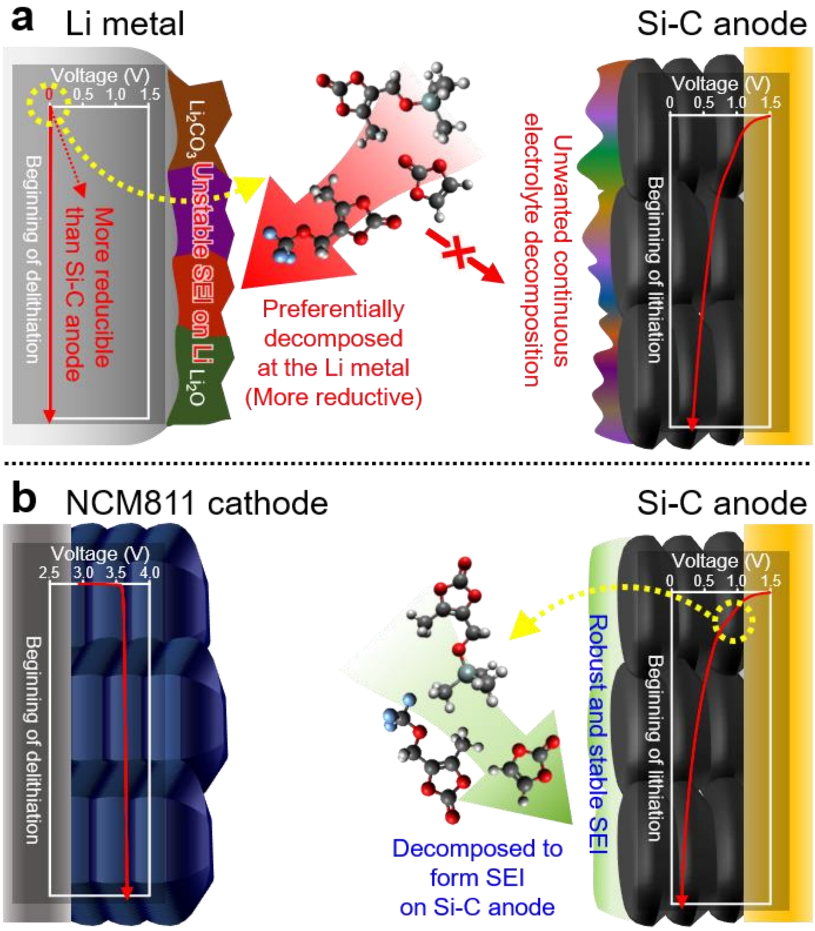

Supplementary Fig. 37. Reduction reaction of VC, DMVC-OCF$_3$, and DMVC-OTMS at the Li metal electrode in the Si-C anode half-cell (**a**) and at the Si-C anode in the NCM811/Si-C full cell (**b**).

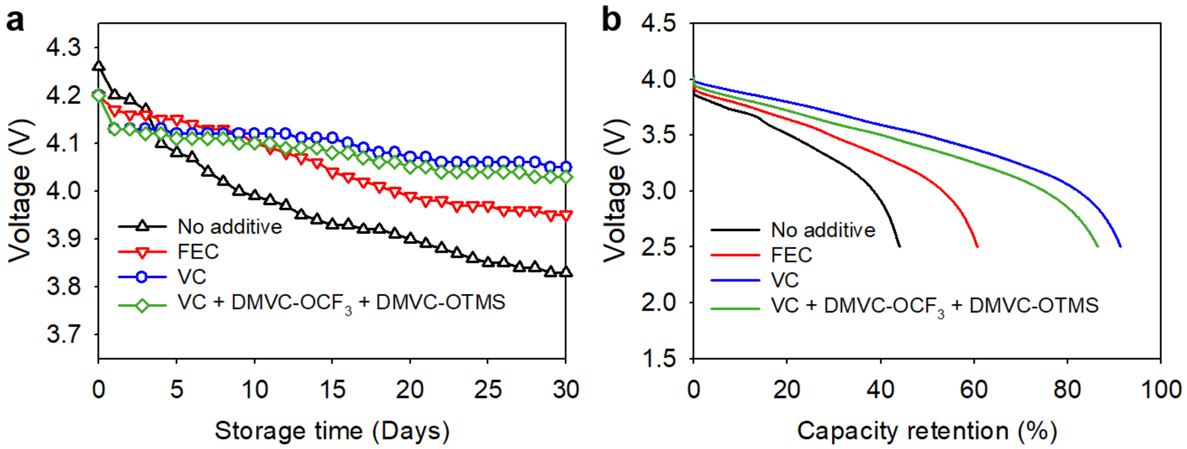

	OCV drop (V)	Capacity retention (%) after 3 days at 60 °C
No additive	0.43	44.0
FEC	0.25	60.7
VC	0.15	91.3
VC + DMVC-OCF_3 + DMVC-OTMS	0.17	86.5

Supplementary Fig. 38. OCV drop (**a**) and capacity retention (**b**) of NCM811/Si-C full cells when stored for 30 days at 60 °C in a fully charged state (SOC 100). NCM811/Si-C full cells with different electrolytes after precycling and standard cycles were charged up to 4.3V at a C/5 rate followed by CV with a C/20 current cutoff at 25 °C and then stored at 60 °C. After 30 days at 60 °C, the capacity retention of full cells was measured at a C/5 rate at 25 °C.

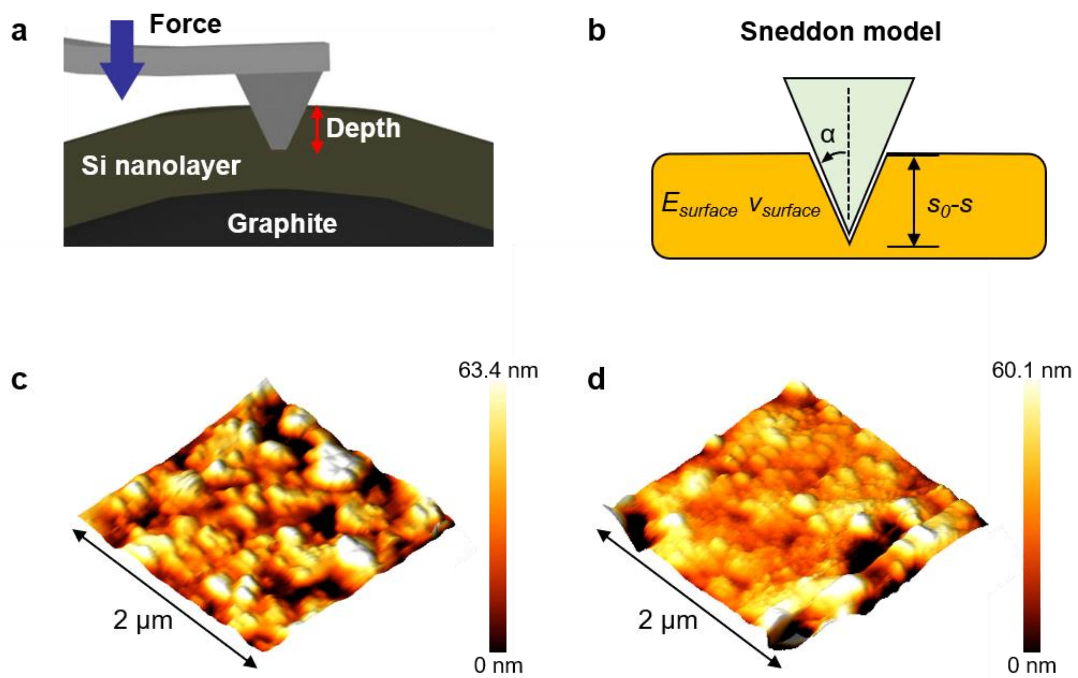

Supplementary Fig. 39. Schematic illustration of the nanoindentation method by AFM (**a**) and the Sneddon *cone-on-flat* model. (**b**). AFM topology images and nanoindentation measurement points of SEI on Si-C anodes after the first cycle with VC-containing electrolyte (**c**) and VC + DMVC-OCF_3 + DMVC-OTMS electrolyte (**d**). Sixteen points on the surface of Si-C anodes cycled with VC, and VC+DMVC-OCF_3+DMVC-OTMS were tipped to measure the mechanical properties of the SEI on the anode.

$$E_{surface} = \frac{\pi}{2}\frac{F_{sneddon}\left(1-\nu_{surface}\right)^2}{(S_0-S)^2\tan\alpha} \qquad \text{(S4)}$$

Equation S4. Young's modulus derivation from FD curves.

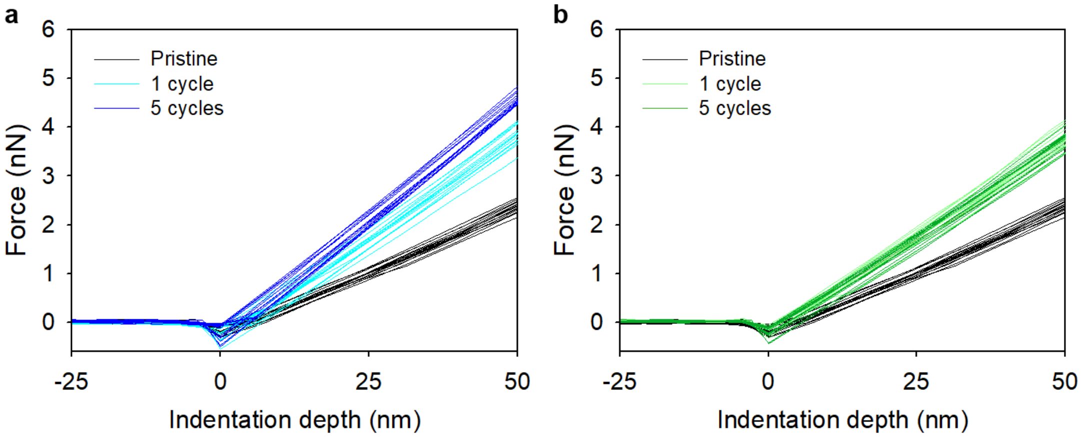

Supplementary Fig. 40. FD curves of Si-C anodes cycled with VC (**a**) and VC + DMVC-OCF$_3$ + DMVC-OTMS (**b**). Black lines represent FD curves of the pristine Si-C anode.

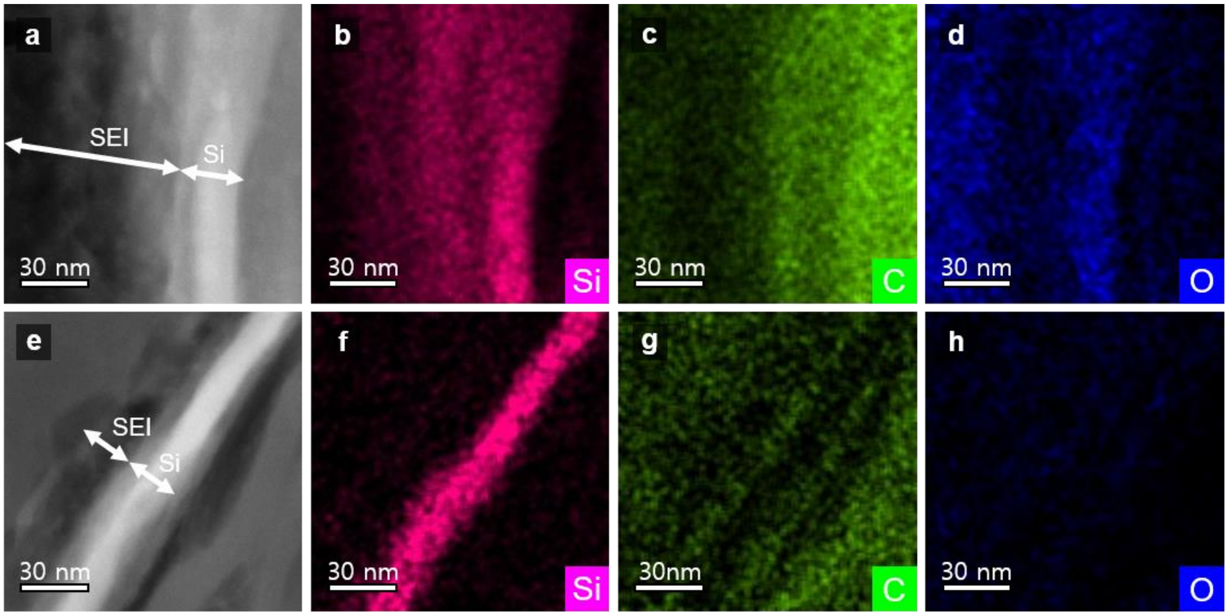

Supplementary Fig. 41. TEM image (**a**) and silicon (**b**), carbon (**c**), and oxygen (**d**) EDS mapping images of the Si-C anode precycled with VC. TEM image (**e**) and silicon (**f**), carbon (**g**), and oxygen (**h**) EDS mapping image of the Si-C anode precycled with VC + DMVC-OCF_3 + DMVC-OTMS.

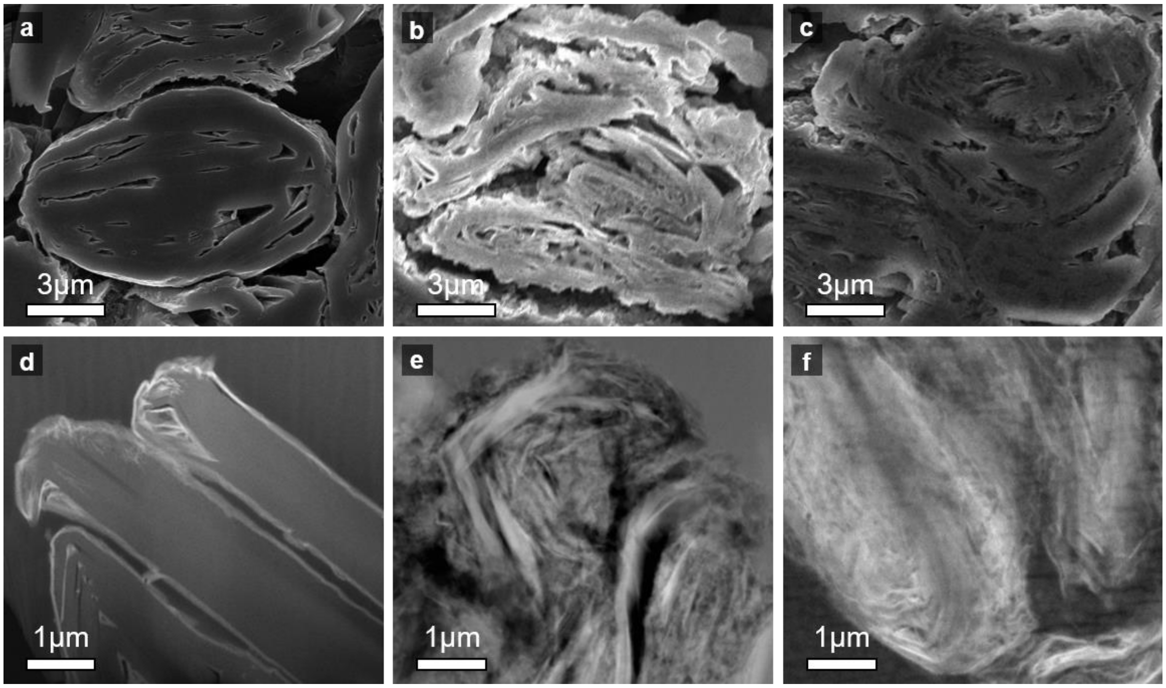

Supplementary Fig. 42. Cross-sectional SEM image of pristine Si-C anode (**a**) and Si-C anodes cycled with VC (**b**) and VC + DMVC-OCF$_3$ + DMVC-OTMS (**c**). TEM image of pristine Si-C anode (**d**) and Si-C anodes cycled with VC (**e**) and VC + DMVC-OCF$_3$ + DMVC-OTMS (**f**).

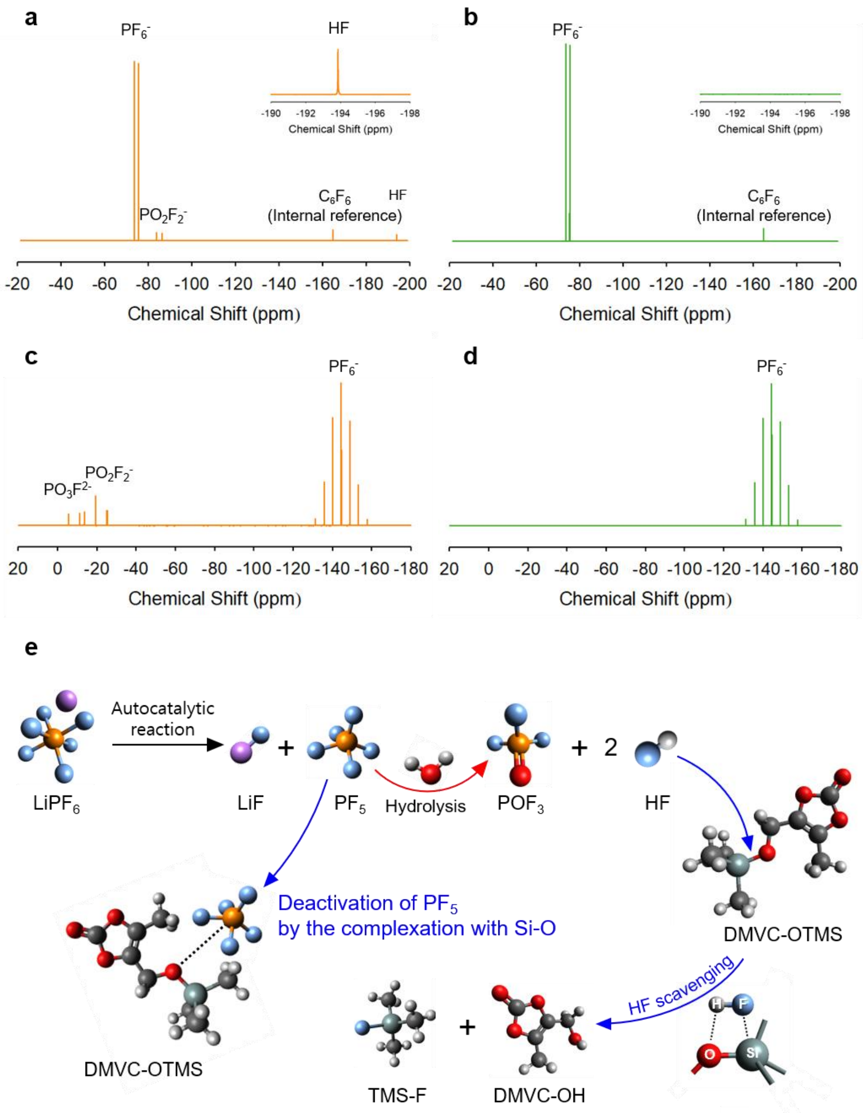

Supplementary Fig. 43. ^{19}F NMR spectra of electrolyte without (**a**) and with 1% DMVC-OTMS (**b**). ^{31}P NMR spectra of electrolyte without (**c**) and with 1% DMVC-OTMS (**d**) after 1 day after adding 1% H_2O. Schematic mechanism of HF scavenging and the PF_5 stabilization effect of DMVC-OTMS (**e**).

Supplementary HRMS and NMR Data

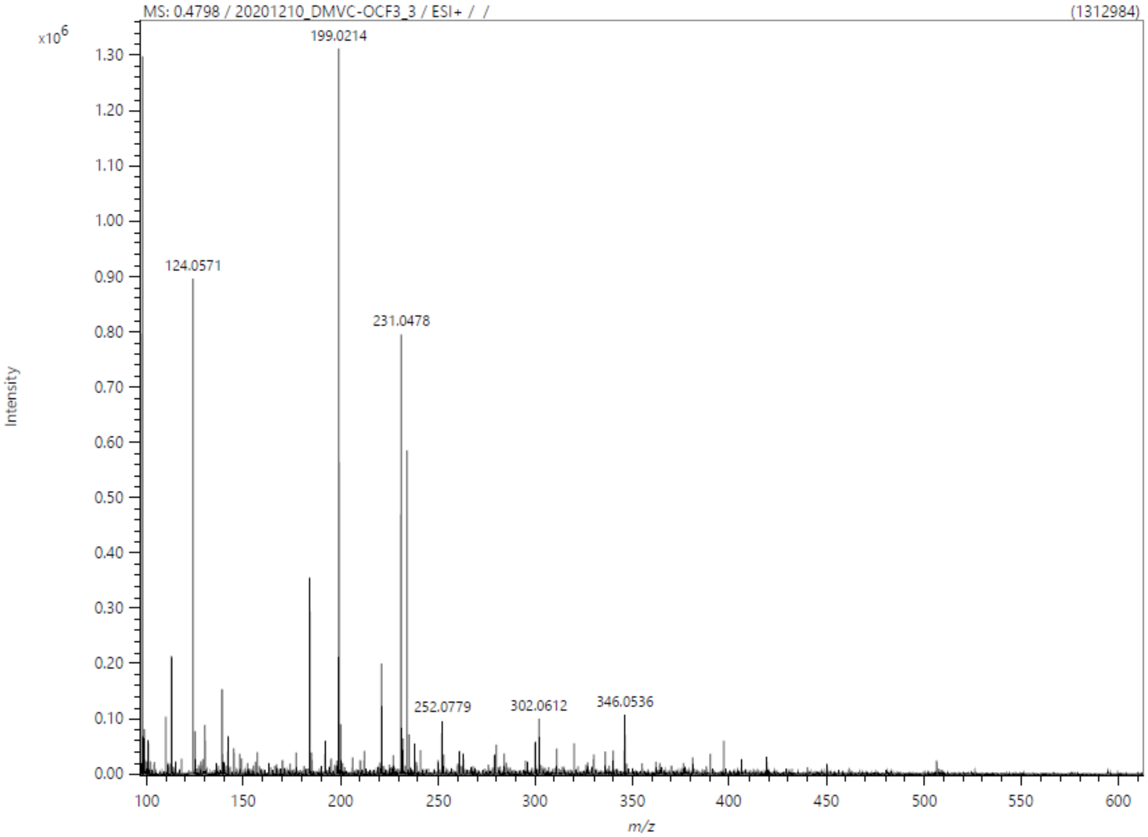

HRMS (ESI+) of DMVC-OCF$_3$

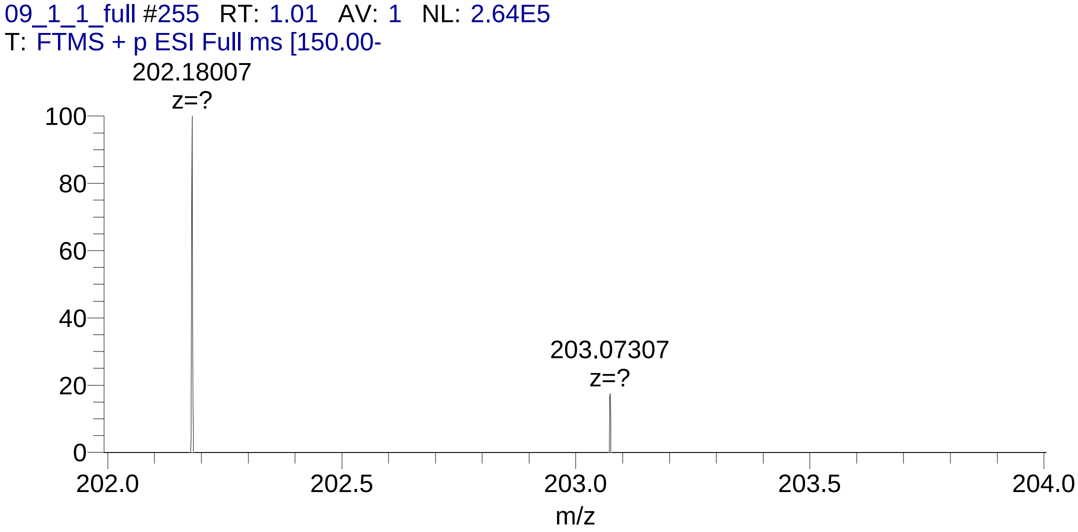

HRMS (ESI+) of DMVC-OTMS

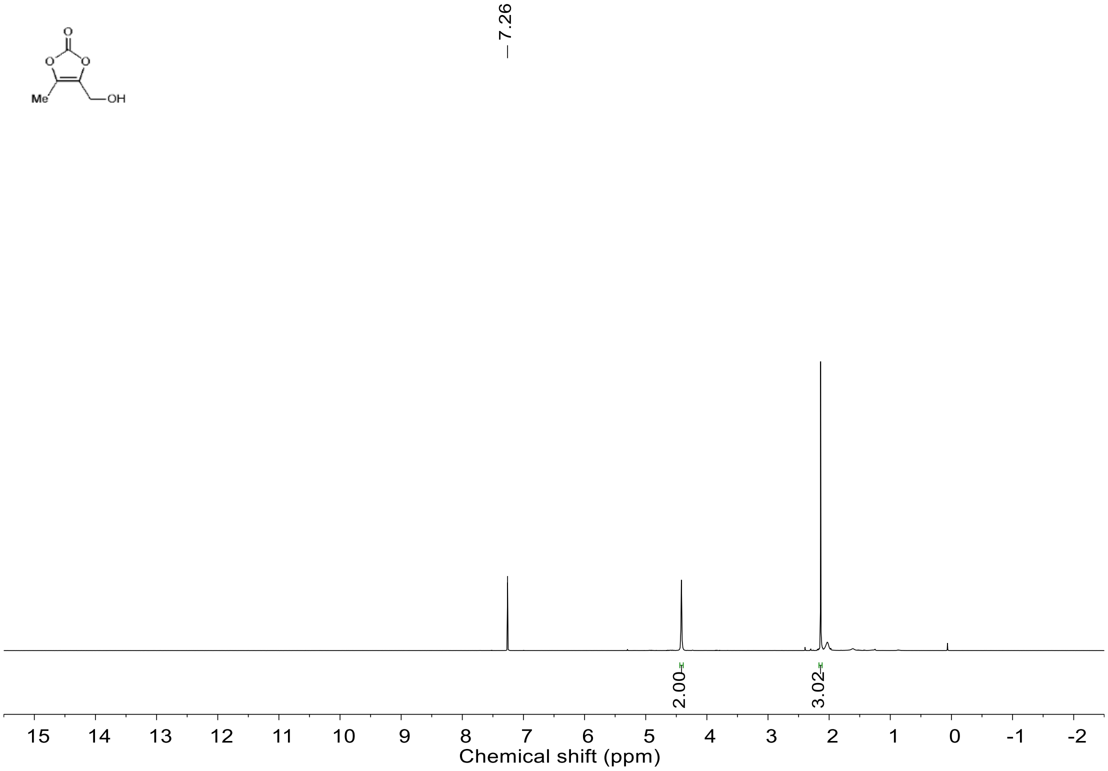

^{1}H NMR (400 MHz, CDC1$_3$) of DMVC-OH

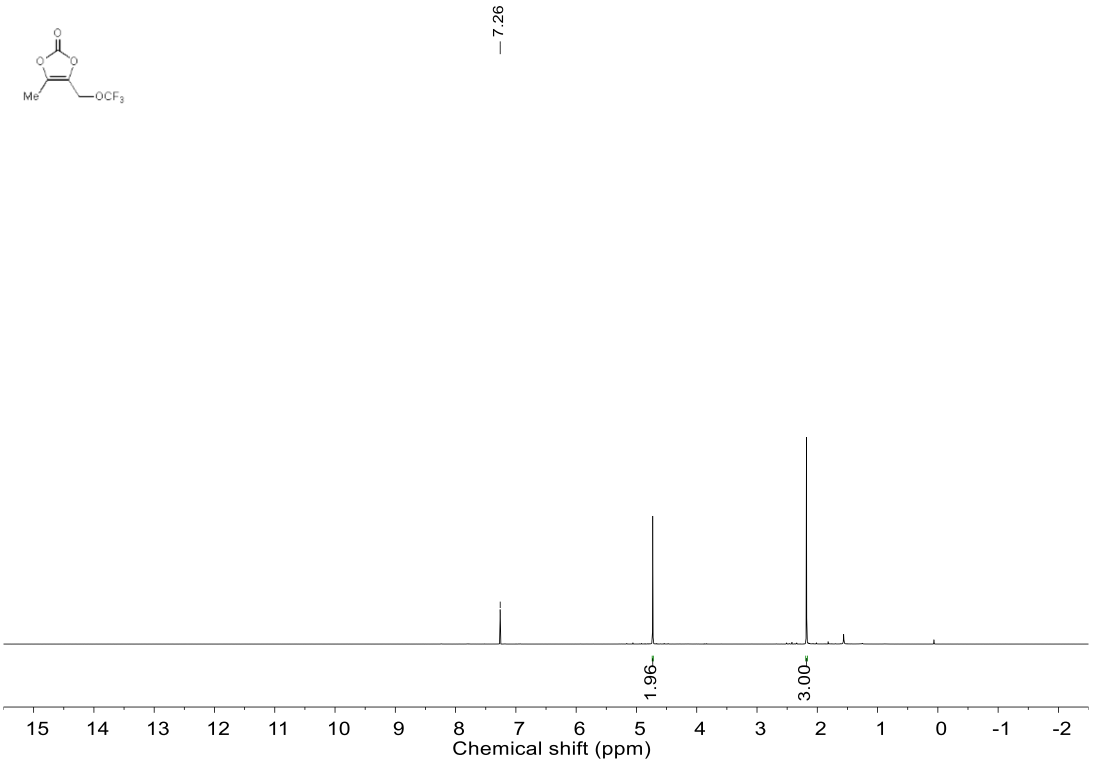

^{1}H NMR (400 MHz, $CDC1_3$) of DMVC-OCF_3

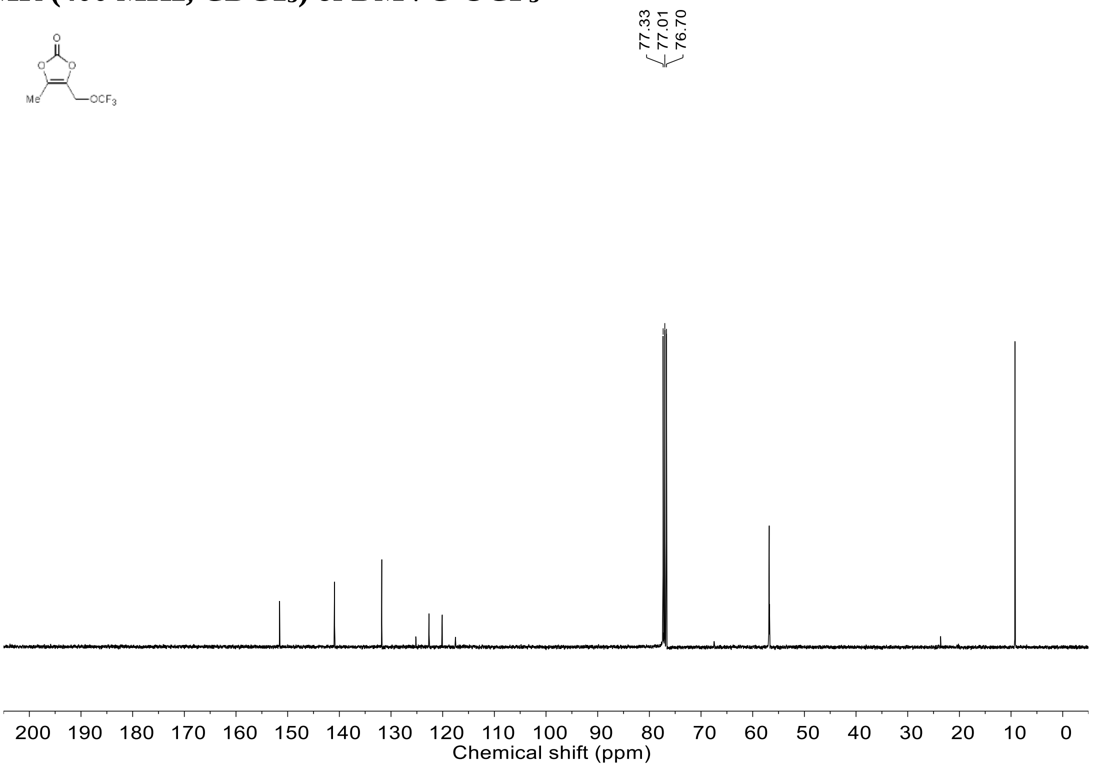

^{13}C NMR (101 MHz, $CDC1_3$) of DMVC-OCF_3

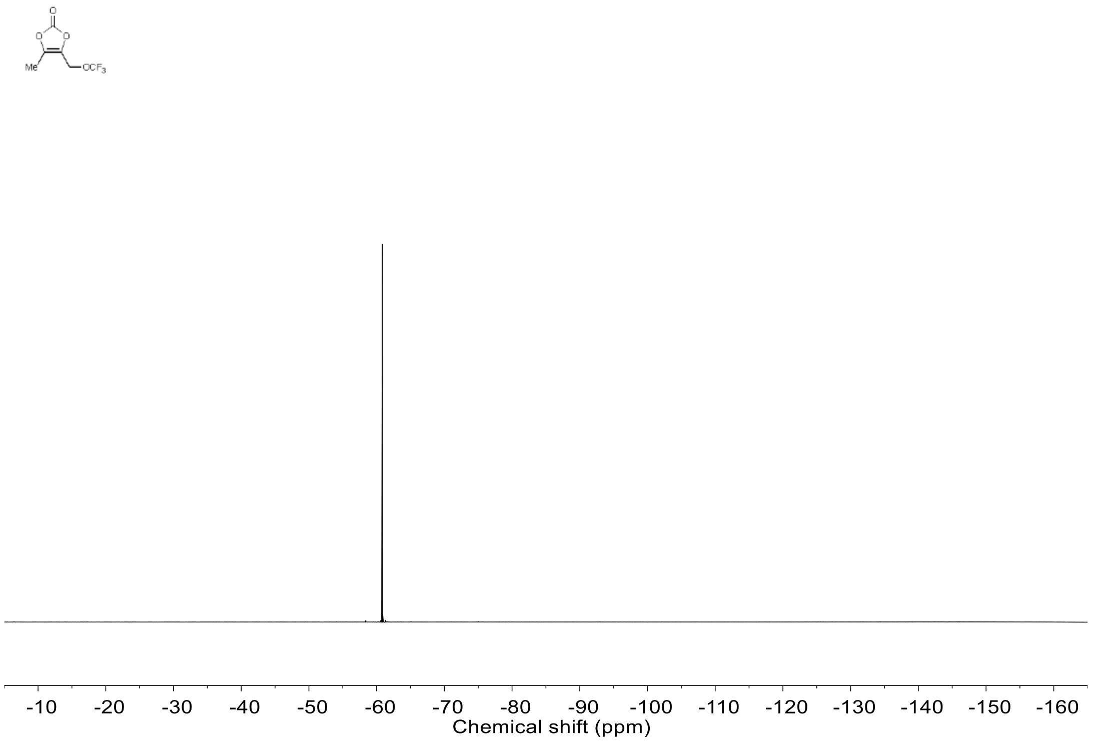

^{19}F NMR (377 MHz, $CDCl_3$) of DMVC-OCF_3

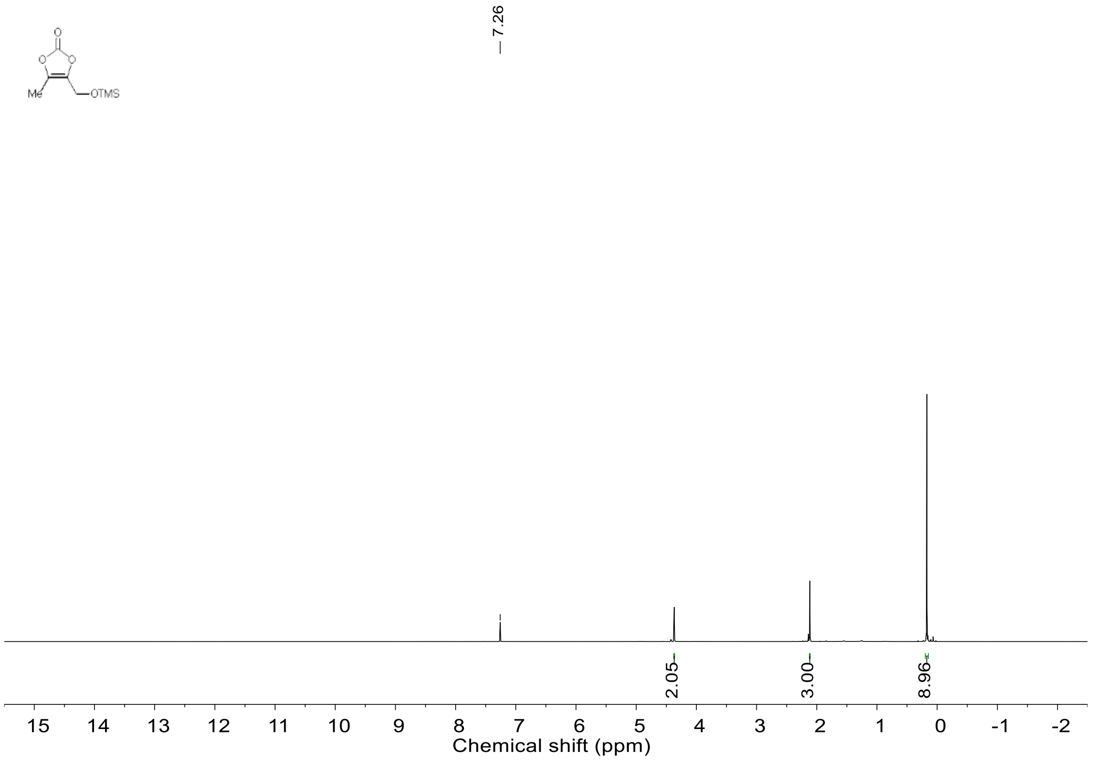

^{1}H NMR (400 MHz, $CDCl_3$) of DMVC-OTMS

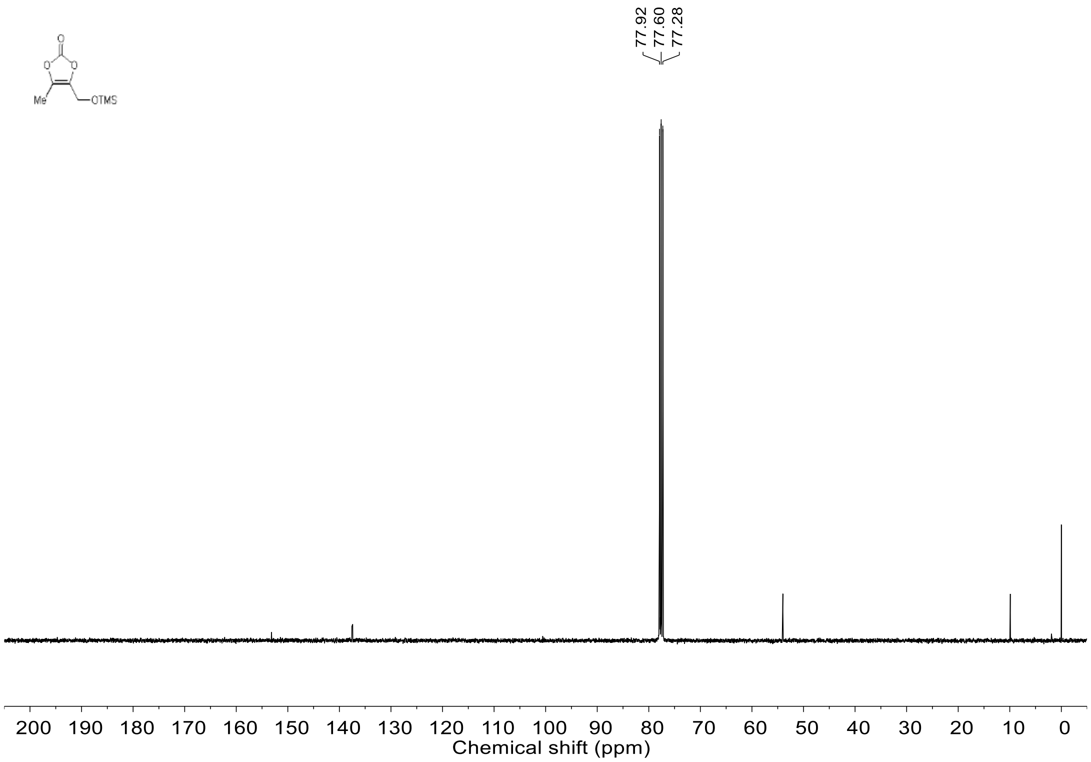

^{13}C NMR (101 MHz, CDC1$_3$) of DMVC-OTMS

Supplementary References

1. Alpegiani, M., Zarini, F., & Perrone, E. On the preparation of 4-hydroxymethyl-5-methyl-1,3-dioxol-2-one. *Synth. Commun.* **22**, 1277-1282 (1992).
2. Becke, A. D. Density-functional thermochemistry. III. The role of exact exchange. *J. Chem. Phys.* **98,** 5648–5652 (1993).
3. Stephens, P. J., Devlin, F. J., Chabalowski, C. F. & Frisch, M. J. Ab initio calculation of vibrational absorption and circular dichroism spectra using density functional force fields. *J. Phys. Chem.* **98,** 11623–11627 (1994).
4. Tkatchenko, A. & Scheffler, M. Accurate molecular van der waals interactions from ground-state electron density and free-atom reference data. *Phys. Rev. Lett.* **102,** 073005 (2009).
5. Mulliken, R. S. Electronic population analysis on LCAO–MO molecular wave functions. I. *J. Chem. Phys.* **23,** 1833–1840 (1955).
6. Mulliken, R. S. Electronic population analysis on LCAO–MO molecular wave functions. II. Overlap populations, bond orders, and covalent bond energies. *J. Chem. Phys.* **23,** 1841–1846 (1955).
7. Halgren, T. A. & Lipscomb, W. N. The synchronous-transit method for determining reaction pathways and locating molecular transition states. *Chem. Phys. Lett.* **49,** 225–232 (1977).
8. Govind, N., Petersen, M., Fitzgerald, G., King-Smith, D. & Andzelm, J. A generalized synchronous transit method for transition state location. *Comput. Mater. Sci.* **28,** 250–258 (2003).
9. BIOVIA Inc. *Materials Studio 2019*. BIOVIA Inc. (2019).
10. Sun, H. *et al.* COMPASS II: extended coverage for polymer and drug-like molecule databases. *J. Mol. Model.* **22,** 47 (2016).
11. Perdew, J. P., Burke, K. & Ernzerhof, M. Generalized gradient approximation made simple. *Phys. Rev. Lett.* **77**, 3865–3868 (1996).
12. Monkhorst, H. J. & Pack, J. D. Special points for Brillouin-zone integrations. *Phys. Rev. B* **13**, 5188–5192 (1976).
13. Xu, W., Vegunta, S. S. S. & Flake, J. C. Surface-modified silicon nanowire anodes for lithium-ion batteries. *J. Power Sources* **196,** 8583–8589 (2011).

14. Zhang, J. *et al.* Direct observation of inhomogeneous solid electrolyte interphase on MnO anode with atomic force microscopy and spectroscopy. *Nano Lett.* **12,** 2153–2157 (2012).

15. Sneddon, I. N. The relation between load and penetration in the axisymmetric boussinesq problem for a punch of arbitrary profile. *Int. J. Eng. Sci.* **3,** 47–57 (1965).

16. Domke, J. & Radmacher, M. Measuring the elastic properties of thin polymer films with the atomic force microscope. *Langmuir* **14,** 3320–3325 (1998).

17. Liu, H. *et al.* Understanding the roles of tris(trimethylsilyl) phosphite (TMSPi) in $LiNi_{0.8}$ $Mn_{0.1}$ $Co_{0.1}$ O_2 (NMC811)/silicon–graphite (Si–Gr) lithium-ion batteries. *Adv. Mater. Interfaces* **2020,** 2000277 (2020).

18. Shan, J. *et al.* Promoting Si-graphite composite anodes with SWCNT additives for half and NCM811 full lithium ion batteries and assessment criteria from an industrial perspective. *Front. Energy* **13,** 626–635 (2019).

19. Xiao, C. *et al.* Walnut-structure Si–G/C materials with high coulombic efficiency for long-life lithium ion batteries. *RSC Adv.* **8,** 27580–27586 (2018).

20. Iqbal, A. *et al.* Lithium-ion full cell with high energy density using nickel-rich $LiNi_{0.8}Co_{0.1}Mn_{0.1}O_2$ cathode and SiO−C composite anode. *Int. J. Miner. Metall. Mater.* **25,** 1473−1481 (2018).

21. Li, J. Y. *et al.* Rational design of robust Si/C microspheres for high-tap-density anode materials. *ACS Appl. Mater. Interfaces* **11,** 4057–4064 (2019).

22. Guo, J. *et al.* Artificial solid electrolyte interphase modified porous SiO composite as anode material for lithium ion batteries. *Solid State Ion.* **347,** 115272 (2020).

23. Aupperle, F. *et al.* The role of electrolyte additives on the interfacial chemistry and thermal reactivity of si-anode-based Li-ion battery. *ACS Appl. Energy Mater.* **2,** 6513–6527 (2019).

24. Liu, Q. *et al.* Stabilized electrode/electrolyte interphase by a saturated ionic liquid electrolyte for high-voltage NMC532/Si-graphite cells. *ACS Appl. Mater. Interfaces* **12,** 23035–23045 (2020).

25. Nguyen, D. T., Kang, J., Nam, K. M., Paik, Y. & Song, S. W. Understanding interfacial chemistry and stability for performance improvement and fade of high-energy Li-ion battery of $LiNi_{0.5}Co_{0.2}Mn_{0.3}O_2$//silicon-graphite. *J. Power Sources* **303,** 150–158 (2016).

26. Budi, A. *et al.* Study of the initial stage of solid electrolyte interphase formation upon chemical reaction of lithium metal and *N*-methyl-*N*-propyl-pyrrolidinium-bis(fluorosulfonyl)imide. *J. Phys. Chem. C* **116**, 19789–19797 (2012).
